# Identification and Characterization of MicroRNAs in Channel Catfish (*Ictalurus punctatus*) by Using Solexa Sequencing Technology

**DOI:** 10.1371/journal.pone.0054174

**Published:** 2013-01-16

**Authors:** Zhiqiang Xu, Jiaping Chen, Xuguang Li, Jiachun Ge, Jianlin Pan, Xiaofeng Xu

**Affiliations:** 1 College of Life Sciences, Nanjing Normal University, Nanjing, China; 2 Freshwater Fisheries Research Institute of Jiangsu Province, Nanjing, China; 3 Department of Epidemiology and Biostatistics, School of Public Health, Nanjing Medical University, Nanjing, China; Colorado State University, United States of America

## Abstract

Channel catfish (*Ictalurus* spp.) is an economically important species in freshwater aquaculture around the world and occupies a prominent position in the aquaculture industry of the United States. MicroRNAs (miRNAs) play important roles in the regulation of almost every biological process in eukaryotes; however, there is little information available concerning miRNAs in channel catfish. In this study, a small-RNA cDNA library was constructed from 10 tissues of channel catfish, and Solexa sequencing technology was used to perform high-throughput sequencing of the library. A total of 14,919,026 raw reads, representing 161,288 unique sequences, were obtained from the small-cDNA library. After comparing the small RNA sequences with the RFam database, 4,542,396 reads that represent 25,538 unique sequences were mapped to the genome sequence of zebrafish to perform distribution analysis and to screen for candidate miRNA genes. Subsequent bioinformatic analysis identified 237 conserved miRNAs and 45 novel miRNAs in the channel catfish. Stem-loop RT-PCR was applied to validate and profile the expression of the novel miRNAs in 10 tissues. Some novel miRNAs, such as ipu-miR-129b, ipu-miR-7562 and ipu-miR-7553, were expressed in all tissues examined. However, some novel miRNAs appear to be tissue specific. Ipu-miR-7575 is predominantly expressed in stomach. Ipu-miR-7147 and ipu-miR-203c are highly expressed in heart, but are relatively weakly expressed in other tissues. Based on sequence complementarity between miRNAs and mRNA targets, potential target sequences for the 45 novel miRNAs were identified by searching for antisense hits in the reference RNA sequences of the channel catfish. These potential target sequences are involved in immune regulation, transcriptional regulation, metabolism and many other biological functions. The discovery of miRNAs in the channel catfish genome by this study contributes to a better understanding of the role miRNAs play in regulating diverse biological processes in fish and vertebrates.

## Introduction

Channel catfish (*Ictalurus spp.*) is an economically important species in freshwater aquaculture around the world and occupies a prominent position in the aquaculture industry of the United States [1]. In recent years, intensive studies of this species, especially concerning disease control, nutritional requirements, immune mechanism and other aspects, have been performed using molecular techniques [2]–[4]. However, little information is available concerning channel catfish microRNAs (miRNAs), which play important roles in the regulation of almost every biological process in eukaryotes. miRNAs are a distinct class of endogenous, non-protein coding RNA molecules about 22 nucleotides in length [5]. Since the discovery of the first miRNAs in *Caenorhabditis elegans* in 1993, thousands of mature miRNAs have been discovered in a wide variety of organisms, including animals, plants and even viruses [6]–[8]. This suggests that miRNAs appeared early in eukaryotic evolution and play fundamental roles in the regulation of gene expression. It is well known that miRNAs play important regulatory roles by targeting mRNAs for cleavage or translational repression. A multitude of studies have discovered critical regulatory functions of miRNAs in many physiological activities, including cell growth, differentiation and apoptosis [9]–[10].

In the past few years, direct cloning methods, bioinformatic approaches and northern blot analyses have been widely used to detect and identify miRNAs [11]–[14]. Many conserved miRNAs have been discovered by traditional methods, such as direct cloning. However, non-conserved miRNAs are seldom identified by traditional methods, because they are often expressed at a lower level than the conserved miRNAs. This explains why small-scale sequencing mainly reveals conserved miRNAs [11]. Recently, next-generation sequencing (NGS) technologies, including Illumina GA (Genome Analyzer) and the ABI SOLiD system, have been developed as powerful sequencing platforms for genomic and transcriptomic studies [15]–[16]. Small RNAs with lower copy numbers can also be detected and profiled owing to high-sensitivity and high-throughput sequencing datasets. With the widespread application of high-throughput sequencing technologies, non-conserved or weakly expressed miRNAs, and many species-specific miRNAs have been identified from diverse organisms [15]–[19].

In recent years, intensive research has revealed the characteristics and functions of miRNAs in many eukaryotes including fish [16]–[17]. Fish represent approximately half of all vertebrate species, and previous studies have identified many miRNAs from model fish species, including zebrafish (*Danio rerio*), fugu (*Takifugu rubripes*), green pufferfish (*Tetraodon nigroviridis*) and medaka (*Oryzias latipes*) [20]–[22]. The identification of miRNAs in aquaculture fish species began with a description of miRNA expression profiles in rainbow trout (*Oncorhynchus mykiss*) by Ramachandra *et al*. [11]. Subsequently, a number of miRNAs have been identified in other aquaculture species, including Japanese flounder (*Paralichthys olivaceus*), bighead carp (*Hypophthalmichthys nobilis*), silver carp (*Hypophthalmichthys molitrix*) and common carp (*Cyprinus carpio L.*) [16]–[18].

Channel catfish have also been used for many years as a model species of lower vertebrates for various biological studies [23]–[24]. Given that miRNAs play key roles by targeting mRNAs for cleavage or translational repression, studies on the characteristics of miRNAs in catfish are particularly useful for functional genome research in this aquaculture species. Recently, Barozai has identified 73 conserved miRNAs in channel catfish by searching an expressed sequence tag (EST) database with bioinformatic methods [13]. Here, we adopted the Solexa GAII platform (Illumina Genome Analyzer) with an integrative bioinformatics strategy to detect and analyze the whole miRNA transcriptome of channel catfish. Using these methods, we have identified 237 conserved miRNAs and 45 novel miRNAs in channel catfish, most of which have more than one isomiR. Subsequently, we validated and profiled the expression patterns of all novel miRNAs in 10 tissues and identified some miRNAs with tissue-specific expression. Moreover, potential targets of the novel miRNAs have been predicted from the reference RNA sequences of the channel catfish. The discovery in this study of miRNA genes in the channel catfish genome will contribute to a better understanding of the roles miRNAs play in regulating diverse biological processes in fish and vertebrates.

## Materials and Methods

### Experimental Animal Collection and RNA Isolation

All animal experiments were performed in accordance with the recommendations in the *Guide for the Care and Use of Laboratory Animals of China*. The study was approved by the Ethics Committee of the Freshwater Fisheries Research Institute of Jiangsu Province.

Healthy channel catfish fingerlings were provided by the National Research Center for *Ictalurus Punctatus* Breeding, China. To obtain whole miRNA transcriptomes, total RNAs from 10 tissues (liver, gill, head kidney, spleen, heart, brain, muscle, stomach, intestines and skin) were extracted using TRIzol reagent (Invitrogen, Carlsbad, CA, USA). Tissues were collected separately from three individuals. After the quality of RNA was assessed by electrophoresis and ultraviolet spectrophotometry, equal concentrations of RNAs from the different tissues were pooled together.

### High Throughput Sequencing

Small RNAs of 16–30 nt in length were first isolated from the total RNA by size fractionation in a 15% TBE urea polyacrylamide gel, and these small RNAs were ligated to an activated 5′ adaptor. Then a 3′adaptor was ligated to the small RNA-5′ adaptor, and reverse transcription PCR using the RT primer was used to create cDNA constructs. Subsequently, a PCR reaction was performed using primers complementary to the two adaptors. The amplified cDNA constructs were purified and sequenced by *Shanghai Majorbio Bio-pharm Technology* using Solexa technology.

### Sequence Data Analysis

Initial reads obtained from Solexa sequencing were processed by summarizing data production, evaluating sequencing quality, calculating the length distribution of small RNA reads, removing low quality reads and adaptor sequences. The remaining clean reads were analyzed by BLAST against the Rfam (ftp.sanger.ac.uk/pub/databases/Rfam) database to annotate rRNA, tRNA, snRNA and other ncRNA sequences. The selected sequences were mapped to the zebrafish genome with a tolerance of one mismatch in the seed sequence. A distribution analysis was performed and candidate miRNA sites were screened out using bowtie software (http://bowtie-bio.sourceforge.net/index.shtml). Subsequently, the candidate sequences were analyzed by miRDeep 2 against all known miRNAs and zebrafish miRNA precursors (miRBase 18.0) (http://www.mdc-berlin.de/en/research/research_teams/systems_biology_of_gene_regulatory_elements/projects/miRDeep/). Sequences which were identical or related (no more than one mismatch) to the reference mature miRNAs were identified as conserved channel catfish miRNAs. The Solexa sequences that can form duplex-like miRNA: miRNA* pairs and map to the conserved miRNA precursors were considered as channel catfish miRNA*s. To further study the conservation of the miRNA*s identified in the present study, multiple sequence alignments were done with miRNA precursors of several typical animals including channel catfish. Subsequently, the sequences that are not identical to the conserved miRNAs were used to BLAST against the zebrafish genome to identify novel miRNAs. To perform deep mining of the dataset, we further used channel catfish EST and genome survey sequences (GSS) databases to identify additional novel miRNAs. For each mapped sequence, hairpin folding was evaluated by sequence analysis to identify the presence of a stem loop with 18 or more base pairs, and the folding energy was calculated by RNAfold (http://www.tbi.univie.ac.at/RNA/).

### Novel miRNA Expression Patterns in Channel Catfish

Quantitative stem-loop RT-PCR with SYBR Green PCR Master Mix (Applied Biosystems) was performed to profile the expression levels of the novel miRNAs in 10 tissues. Primers for stem-loop RT-PCR were designed according to descriptions in prior studies (Table S1) [25]. Relative expression levels of the 45 novel miRNAs were measured in terms of threshold cycle value (Ct) and were normalized to 5S rRNA using the equation 2*^−ΔCt^*, in which *ΔCt = Ct_miRNA_–Ct_5s_*. All real-time reactions were run using the ABI PRISM 7900 HT platform (Applied Biosystems,Inc.) and performed in triplicate. Specificity of amplification for each transcript was confirmed by melting curve analysis using SDS software (Applied Biosystems,Inc.). A tissue expression heatmap of the channel catfish miRNAs and hierarchical clustering for both tissues and miRNAs were constructed using Multiexperiment Viewer (MeV) software (http://www.tm4.org/mev.html).

### Prediction of Potential miRNA Targets in Channel Catfish

Potential target sequences for the newly identified miRNAs were predicted using the same strategy described in prior studies [13]. First, novel miRNAs identified in the present study were used to search for antisense hits in the reference RNA sequences (refseq_rna) of the channel catfish (taxid: 7998). Subsequently, to predict the target sequences, mRNA sequences exhibiting perfect or near perfect complementarity with corresponding miRNAs were selected and analyzed with RNAhybrid, a miRNA target detection software [26].

## Results and Discussion

### Solexa Sequencing of Small RNAs

**Figure 1:**
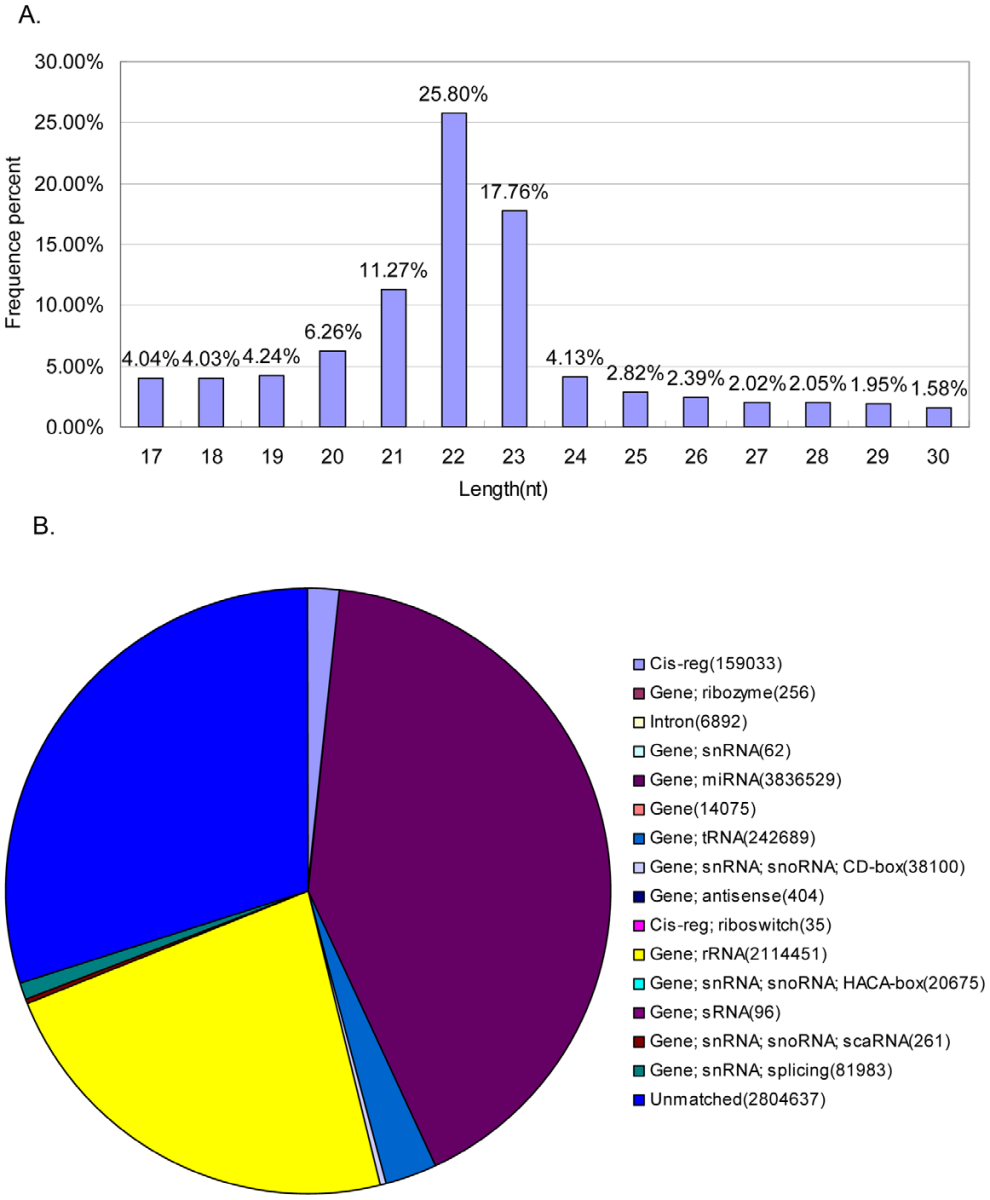
Length distribution and annotation of small RNAs derived from Solexa sequencing of channel catfish small RNAs. A: Length distribution of sequenced small RNAs; B: Clean reads were BLAST searched against the RFam 10.1 database to annotate rRNAs, tRNAs, snoRNAs and other snRNAs.

To increase the coverage of channel catfish miRNAs, a library of small cDNAs was generated with pooled total RNAs and subjected to Solexa high-throughput sequencing. 14,919,026 raw reads, representing 161,288 unique sequences, were obtained. The length distribution analysis showed that 54.8% of reads were 21–23 nucleotides (nt) (Figure 1A). After removal of the 5′ and 3′ adapter sequences, sequences longer than 26 nt or shorter than 15 nt, and unique sequences with fewer than two copies, 9,320,178 high quality clean reads were extracted for subsequent analysis. After comparing the small RNA sequences with the RFam 10.1 database, 2,679,012 reads of rRNA, tRNA, snoRNA and other snRNAs were annotated and removed (Figure 1B.). The remaining 6,641,166 reads were retained for miRNA analysis.

**Figure 2:**
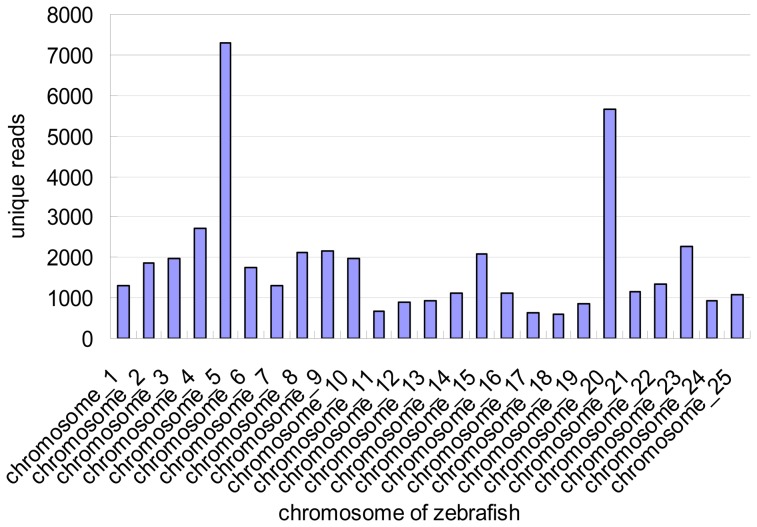
Number and distribution of unique reads mapped to the genome sequence of zebrafish.

Given that no whole genome data for channel catfish are available, we aligned the selected small RNA sequences to the genome sequence of zebrafish (http://hgdownload.cse.ucsc.edu/downloads.html#zebrafish ), which is the closest evolutionarily related species with an available sequenced genome, to perform a distribution analysis on a genomic scale. Among the screened sequences, 4,542,396 reads that represent 25,538 unique sequences were mapped to the genome sequence of zebrafish to screen for candidate miRNA sites (Figure 2). The alignments between the catfish sequences and the genome sequence of zebrafish help the organization of catfish data on a genomic scale. Moreover, assessing conserved synteny between the two species has proved useful in gene isolation and QTL fine-mapping studies [27].

### Conserved and Novel miRNAs in Channel Catfish

To identify conserved miRNAs in channel catfish, we compared the 25,538 unique sequences against mature miRNAs in miRBase 18.0. miRNAs with sequence homologous to known miRNAs are classified as conserved miRNAs. Allowing at most one mismatch between sequences, we identified 237 conserved miRNAs, which belong to 105 families (Table S2). The reads of these miRNAs range from two to 479,533, indicating that not only highly expressed miRNAs but also weakly expressed miRNAs are identified by Solexa sequencing.

The sequences that did not match known miRNA precursors were compared with the zebrafish genome sequence to detect potential novel miRNAs. This allowed us to identify 18 novel miRNAs in channel catfish (Table 1). Since the prediction is based on the zebrafish genome, these miRNAs should theoretically exist in zebrafish as well. Whole genome data for the channel catfish are not yet available; therefore, the approach of using the zebrafish genome to predict novel miRNAs relies on the phylogenetic conservation of the sequence. This means that some novel miRNAs will not be identified. Therefore, to perform deep mining of the dataset, we used channel catfish EST and GSS databases and miRDeep2 software to identify additional novel miRNAs. Twenty-seven miRNA precursors with stem-loop structures that mapped to channel catfish EST and GSS sequences were found (Table 2). It is worthwhile mentioning that most of the novel miRNAs identified in channel catfish are relatively weakly expressed compared with conserved miRNAs. The average number of reads for novel miRNAs is lower than that of conserved miRNAs (148.33 *vs.* 38581.71). The same phenomenon has also been observed for other species [16]–[18], which suggests that novel miRNAs are usually weakly expressed while conserved miRNA genes are highly expressed.

**Table 1:** Novel miRNAs identified in channel catfish with the genome of zebrafish.

miRNA	sequence	read count	position in the mapped reference sequence
ipu-miR-24b	uggcucaguucagcaggaac	748	2∶33623651.33623708:+
ipu-miR-7547	agcggcgucagaagcgauggcc	82	7∶33269277.33269339:+
ipu-miR-101a-3p	caucagcacugugauaacuga	30	6∶31057627.31057683:−
ipu-miR-7147	uguaccaugcugguagccagu	53	24∶10720304.10720362: −
ipu-miR-29a	acugauuuccucugguguu	215	23∶20637186.20637247: −
ipu-miR-16c	ccagcagcacggucaauacug	52	1∶47219524.47219591:+
ipu-miR-199b	uaaccaaugugcagacuacugu	39	5∶1695989.1696054:+
ipu-miR-7548	agccgcggcuguaggagc	192	23∶30968675.30968755:+
ipu-miR-203c	uugaacuguuaagaaccacugc	327	17∶45975697.45975757: −
ipu-miR-551	gcgacccauccuugguuucu	10	15∶34583545.34583600:+
ipu-miR-7549	gaucggugcagcggcggc	291	16∶1499061.1499105: −
ipu-miR-129b	uuugggguaagggcuuccuggcu	4	4∶18115667.18115726:+
ipu-miR-7550	auccggcucgaaggacca	844	13∶37954231.37954286: −
ipu-miR-3618	gauuuccaauaauugagacagu	372	5∶26190297.26190355:+
ipu-miR-7551	ggggccugaguccuucugg	88	6∶52967777.52967859:+
ipu-miR-7552	aaugucccuuaauuguuugguu	85	12∶13216988.13217053: −
ipu-miR-7553	ugacgucauuagcgacccgacc	364	7∶33269276.33269336: −
ipu- miR-7554	acauuuugucuaucugaaa	2	7∶1429428.1429502: −

**Table 2:** Novel miRNAs identified in channel catfish with the EST and GSS database.

miRNA	sequence	read count	position in the mapped reference sequence
ipu-miR-7555	cuccagcccggacacccaggac	54	gi|204069919|gb|FD326800.1|FD326800∶128.187: −
ipu- miR-7556	uacaguaacuugaagacaacgu	38	gi|204084719|gb|FD331302.1|FD331302∶133.194:+
ipu- miR-7557	ugcuugcguucugucuguucccu	27	gi|224241675|gb|GH686669.1|GH686669∶235.295:+
ipu- miR-7558a	ggcugagauugggagcacucccu	14	gi|18646130|gb|BM494949.1|BM494949∶104.155:+
ipu- miR-7559	ugccacacgacugcucagcuca	61	gi|224281223|gb|GH675949.1|GH675949∶558.611:+
ipu- miR-7560	uaccugucugcaaucuugaagc	10	gi|224250215|gb|GH657351.1|GH657351∶527.586:+
ipu- miR-7558b	agcugagauugggagcacacuc	29	gi|224238044|gb|GH682245.1|GH682245∶458.509: −
ipu- miR-7561	ugauucagagucgagcucgcuu	49	gi|204112847|gb|FD367553.1|FD367553∶710.773:+
ipu- miR-7562	cacacacacucaugaacacaca	10	gi|224239931|gb|GH646923.1|GH646923∶14.75: −
ipu- miR-7563a	aaccgcguacuugccuacuaua	7	gi|201030860|gb|FD036996.1|FD036996∶371.455: −
ipu- miR-7563b	accgcguacuuaccuacuguaug	22	gi|204062776|gb|FD116111.1|FD116111∶156.232: −
ipu- miR-7564	uuucagagccagagaucgacagc	6	gi|204284216|gb|FD314225.1|FD314225∶213.281: −
ipu- miR-7563c	accgcguacuuaccuacuguau	26	gi|40575287|gb|CK414333.1|CK414333∶481.563:+
ipu-miR-7565	uuccugcugaacugagccagu	20	gi|204269725|gb|FD364850.1|FD364850∶619.675:+
ipu-miR-7566	ucaucggcucaucagacacgc	29	gi|40573358|gb|CK412696.1|CK412696∶750.807:+
ipu-miR-7567	uugcccagaucgaucgccagcu	5	gi|56167725|gb|CV991851.1|CV991851∶94.162:+
ipu- miR-7568	cugaccgaccaagugcugaau	4	gi|204080548|gb|FD371264.1|FD371264∶583.636: −
ipu- miR-7569	uauaaucuugauguuucucca	26	gi|40572877|gb|CK412282.1|CK412282∶114.188:+
ipu- miR-7570	uucuuaugugcgccggcacucu	2	gi|118496311|gb|EE993615.2|EE993615∶401.459:+
ipu- miR-7571	caggcuacaugacaccacccuga	30	gi|18393650|gb|BM425126.1|BM425126∶50.110:+
ipu- miR-7572	gauugcagcuuuacaguguuucc	3	gi|200944081|gb|FD030799.1|FD030799∶333.394: −
ipu- miR-7573	aggcugagccugauggcacugag	2	gi|204132990|gb|FD262187.1|FD262187∶225.297:+
ipu- miR-7574	uuuaucucuacucgcucgucu	9	gi|204025299|gb|FD323591.1|FD323591∶204.260: −
ipu- miR-7575	gcauggucaugaucaugguc	2	gi|30224238|gb|CB938847.1|CB938847∶656.706:+
ipu- miR-7576	agaacauucaaccgccgcaca	2	gi|204187343|gb|FD336486.1|FD336486∶198.251:+
ipu- miR-7577	cucggacauuuuggacucgga	14	gi|204280683|gb|FD352971.1|FD352971∶10.55: −
ipu- miR-457b	uagcagcacaucaauauuggca	2288	gi|281572797|gb|FI880023.1|FI880023∶146.211: −

Generally speaking, abundant miRNAs play fundamental and broad regulatory functions in maintaining biological processes. The most highly expressed miRNA family in channel catfish was ipu-miR-181 (1,781,434 reads). Studies on these miRNAs in mammals indicate that miR-181 is involved in multiple roles in immune regulation and disease. Li *et al.* found that increasing miR-181a expression in mature T cells augments their sensitivity to peptide antigens, while inhibiting miR-181a expression in immature T cells reduces sensitivity and impairs both positive and negative selection [28]. Cichocki *et al.* demonstrated that nemo-like kinase (NLK), an inhibitor of Notch signaling, is a target of miR-181 in natural killer cell (NK) cells, and knockdown of NLK mirrors the developmental effect of miR-181 overexpression. Therefore, they concluded that miR-181 can promote NK cell development, at least in part, through the suppression of NLK [29]. Let-7 is a highly significant miRNA family that was first discovered in *C. elegans* [30]. Ten members of the let-7 miRNA family were characterized in channel catfish by high throughput sequencing, all of which have a high read frequency (ranging from 2,803 to 168,690). The *C. elegans* let-7 family plays a key role in regulating late developmental events by down-regulating multiple genes that contain 3′ UTR sequences complementary to the seed region. Previous studies in mammals have also indicated that the let-7 gene family can regulate the expression of major cytokine-inducible proteins in response to microbial challenge [31]–[32]. Possibly because of vital roles in channel catfish, ipu-let-7 was identified as the second most abundant miRNA family (1,471,153 reads). Another highly expressed miRNA family was ipu-miR-9 (807,317reads), an important miRNAs that show brain-specific expression in mammals and fish [33]–[34]. Leucht *et al.* found that the organizing activity and progenitor state of the midbrain-hindbrain boundary (MHB) are co-regulated by miR-9 during late embryonic development of zebrafish. Functional studies demonstrate that miR-9 can target several components of the Fgf signaling pathway, thereby promoting the organizing activity of the MHB. In addition, zebrafish miR-9 promotes progression of neurogenesis in the MH, defining the MHB progenitor pool [34].

### Phenomenon of Multiple isomiRs in Channel Catfish

**Figure 3:**
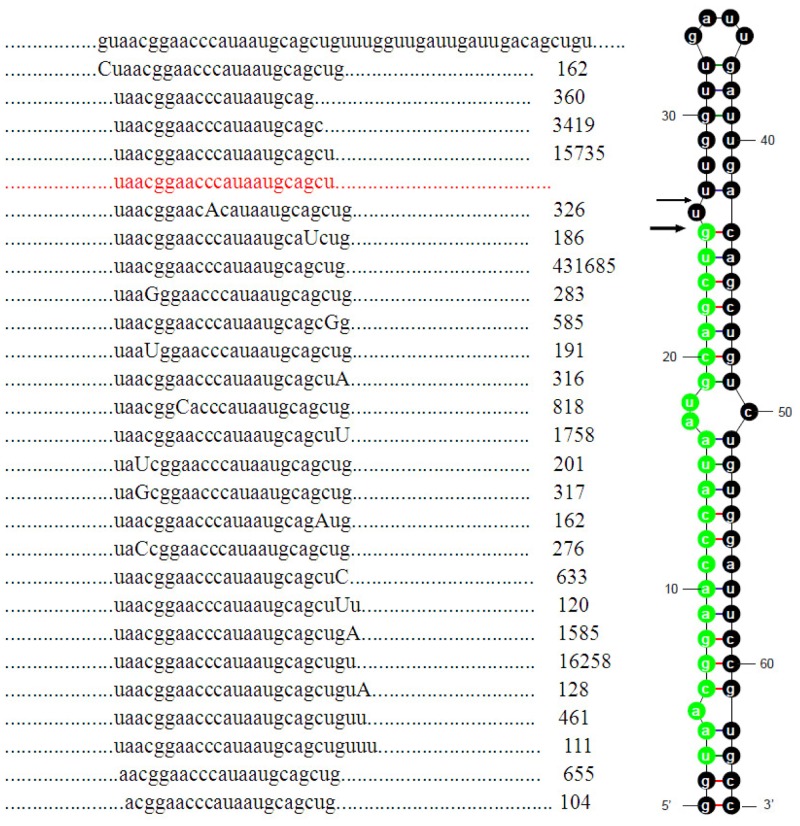
Details of ipu-miR-462 isomiRs including sequence counts. A portion of the miRNA precursor, multiple isomiRs (each with more than 99 counts) with their sequence counts and the pre-miRNA secondary structure with dominant cleavage sites are presented. Single nucleotide substitutions and additional 5′ or 3′ non-template nucleotides are highlighted by capital letters. The reference miRNA sequence from miRBase is shown in red. Inferred dominant cleavage sites are indicated by arrows. Large arrows represent cleavage sites for the most abundant isomiR, whereas small arrows indicate cleavage sites for secondary abundance isomiRs. The most abundant mature miRNAs are indicated by the sequence in green.

Recent studies involving miRNA sequencing with NGS technology have shown the existence of isomiRs, which are encoded by the same pre-miRNAs and exhibit sequence variations from the reference miRNAs in miRBase [35]–[36]. In the present study, the phenomenon of multiple isomiRs was again observed and an isomiR example is illustrated by ipu-miR-462 (Figure 3). Twenty-seven isomiRs (each with more than 99 counts) are detected in ipu-miR-462, which is the most abundant miRNAs in channel catfish. Some isomiRs, detected with single nucleotide substitutions including transition and transversion, may represent the result of pre-miRNA editing. Of the nine single nucleotide substitutions observed, the most prominent were A-to-C (39.6%), C-to-A (17.7%) and A-to-G (11.5%). Another type of detected isomiR has additional 5′ or 3′ non-template nucleotides. Prior studies show that isomiRs with 5′ and/or 3′ non-template additional nucleotides may have shorter, longer or consensus lengths with respect to their canonical miRNA sequences [37]–[38]. The nucleotide most commonly added to ipu-miR-462 at the 3′ end is adenine (2,029 reads), followed by uracil (1,878 reads), then cytidine (633 reads). Multiple isomiRs with various 5′ and/or 3′ ends are thought to be the result of inexact Drosha and Dicer processing. Abundantly expressed isomiRs can be used to estimate dominant cleavage sites of Drosha and Dicer during pre-miRNA processing [39]. The phenomenon of multiple isomiRs, especially for isomiRs with 3′ additions, is widely detected in many species [39]–[41]. Additional 3′ non-template nucleotides in isomiRs may contribute to miRNA stability and may play a key role in miRNA:target interactions. For example, isomiRs with 3′ additions may increase miRNA stability in *Drosophila* and may attenuate the effectiveness of some specific miRNAs [40]–[41]. IsomiRs with additional 3′ non-template nucleotides may also be involved in the pathogenesis of many human diseases [39].

### Conservation of miRNA* in Channel Catfish

The maturation mechanism of miRNAs involves cleavage of the primary transcripts into pre-miRNA by Drosha, followed by processing of the pre-miRNA into a miRNA/miRNA* duplex by Dicer. In most cases, after being cleaved into the miRNA/miRNA* duplex, only one strand of the duplex, from either the 5′ arm or the 3′ arm of the pre-miRNA, is selected as the mature miRNA and incorporated into the RISC complex [5]. It is also observed that both strands of the duplex can contribute simultaneously to two mature miRNAs of equal abundance, named with a -5p or -3p suffix, or of unequal abundance, designated with an asterisk(*) suffix for the minor miRNA [42]. From the 25,538 unique sequences, a total of 120 duplex-like miRNA: miRNA* pairs were obtained, in which the mature miRNAs and miRNA*s align to the 5′ and 3′ end regions of the precursors, respectively. Many studies indicate that, in most cases, only mature miRNAs function in regulating gene expression and the miRNA* sequence is degraded by an unknown mechanism. However, recently, some miRNA* sequences were reported as mature functional miRNAs with abundant expression, and miRNA/miRNA* ratios may vary dramatically in different stages of development [43]. In channel catfish, most of the miRNA*s were detected at low levels except for ipu-miR-144*, ipu-miR-153b* and ipu-miR-16b* (Table S1). Some highly expressed miRNAs, like ipu-miR-462 and ipu-miR-9, were poorly represented by their star sequences. Therefore, the relatively high number of reads of ipu-miR-144*, ipu-miR-153b* and ipu-miR-16b* indicates that they may play a functional role in regulating gene expression. In previous studies, such a phenomenon has also been described in bighead carp and silver carp. A relatively higher number of miR-1388* compared with miR-1388 was detected in bighead carp and silver carp using high-throughput sequencing [16].

**Figure 4:**
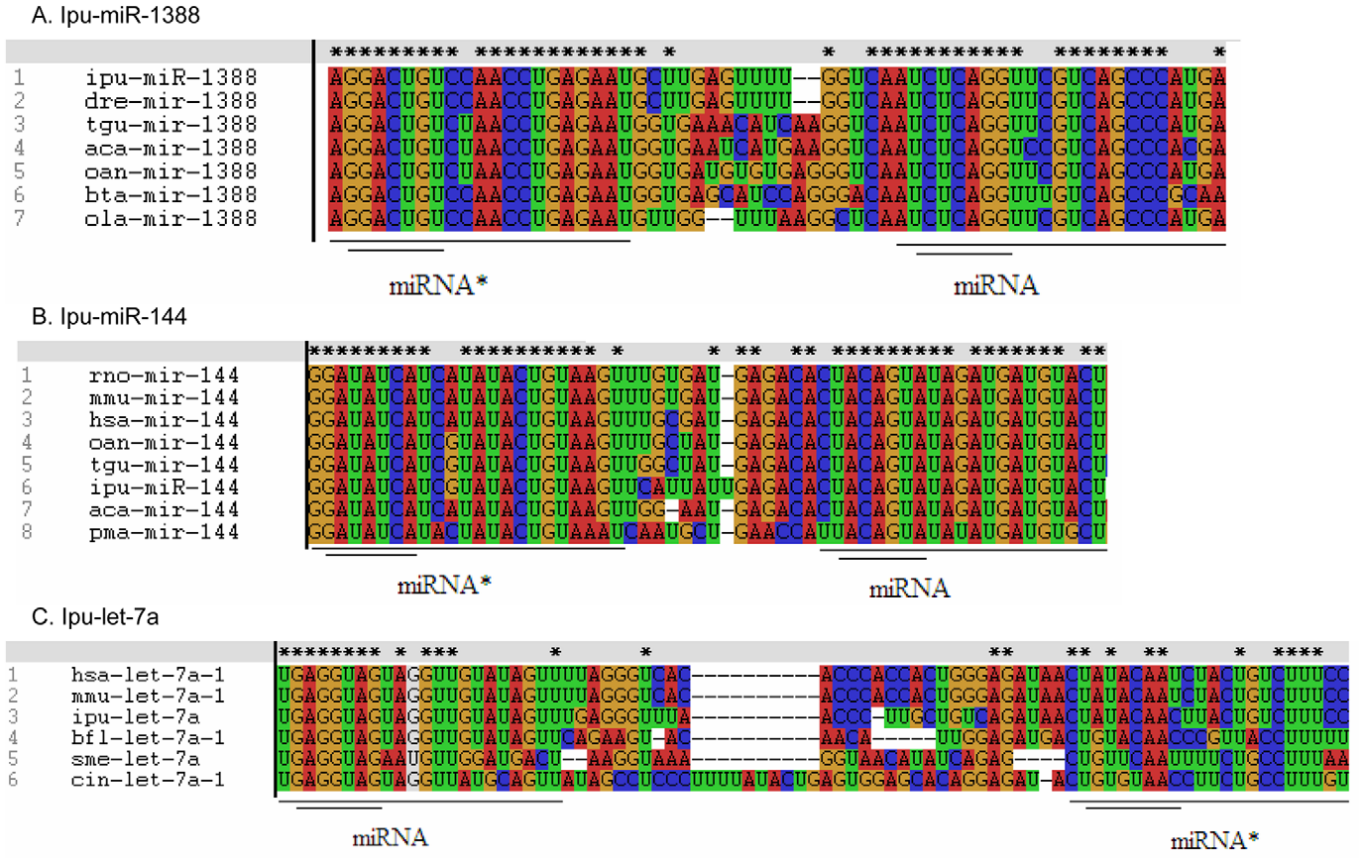
Conservation analysis of miRNA*s identified in channel catfish. MiR-1388 and miR-144 are compared among several vertebrates. Let-7a is compared across vertebrates, protochordates and invertebrates. Alignments were performed using ClustalX. Mature miRNA sequences and their star sequences are single line underlined. Seed regions are double underlined. ipu: *I. punctatus*; has: *H. sapiens*; mmu: *M. musculus*; dre: *D. rerio*; tgu: *T. guttata*; aca: *Anolis carolinensis*; oan: *Ornithorhynchus anatinus*; bta: *Bos taurus*; ola: *O. latipes*; rno: *Rattus norvegicus*; pma: *Petromyzon marinus*; bfl: *B. floridae*; sme: *S. mediterranea*; cin: *C. intestinalis.*

Prior studies have demonstrated important roles for miRNAs and many of them are highly conserved even between vertebrates and invertebrates. However, miRNA*s may not be so highly conserved because although miRNAs and miRNA*s are complementary, their base-pairing is not perfect (for instance, bulges exist and GU pairing is allowed) [16]. We randomly selected typical model animals to analyze the conservation of the miRNA*s identified in channel catfish in vertebrates and invertebrates (Figure 4). Some miRNA* strands, such as ipu-miR-1388* and ipu-miR-144*, are highly conserved in vertebrates. Although divergence patterns of mature miRNAs are different to those of miRNA*s, both are well conserved, especially for the seed sequences (Figure 4 A and B). We selected let-7 in several typical animals to analyze the conservation of the miRNA*s identified in channel catfish across vertebrates and invertebrates. The comparison of let-7a precursors among *Homo sapiens*, *Mus musculus*, *Ciona intestinalis*, *Branchiostoma floridae* and *Schmidtea mediterranea* showed that the seed sequences of the mature miRNAs are highly conserved among the five species. However, the miRNA* sequences, though conserved in vertebrates (*H. sapiens*, *M. musculus* and *I. punctatus*), are less conserved among protochordates (*C. intestinalis* and *B. floridae*) and invertebrates (*S. mediterranea*) even in their seed sequences (Figure 4C). Therefore, during evolution of miRNA genes, functional mature miRNAs have remained well conserved especially in their seed sequences, while miRNA* sequences show various evolutionary patterns in different species.

### Novel miRNA Expression Patterns in Different Channel Catfish Tissues

**Figure 5:**
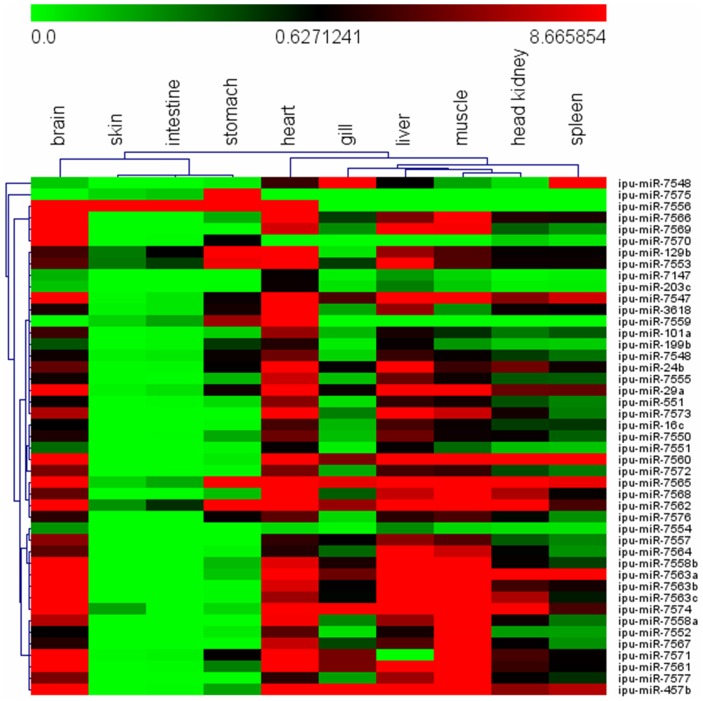
Heat map showing newly identified miRNA expression patterns in 10 tissues (liver, gill, head kidney, spleen, heart, brain, muscle, stomach, intestines and skin) measured by stem-loop RT-PCR. Relative expression levels of the 45 novel miRNAs were measured in terms of threshold cycle value (Ct) and were normalized to 5S rRNA. The expression data were analyzed by hierarchical clustering for both tissues and genes.

Owing to financial restraints and a desire to obtain the whole miRNA transcriptome of channel catfish, the cDNA library of small RNAs was constructed with pooled total RNAs from different tissues. Therefore, we did not obtain tissue-specific expression profiles of miRNAs. In general, the tissue distribution of miRNAs provides an essential baseline reference to analyze variation of miRNA expression under various physiological conditions. Quantifying miRNAs in different tissues is an important initial step to investigate the functions of miRNAs. The limitations of short length and low expression levels of miRNAs necessitate specific and sensitive methods for their detection. Stem-loop RT-PCR, using primers with stem-loop structures, has become one of the most reliable and sensitive experimental approaches for miRNA validation. We, therefore, used stem-loop RT-PCR to validate and profile the expression of the novel miRNAs in different tissues. Ten channel catfish tissues including brain, heart, skin, intestines, stomach, gill, liver, muscle, head kidney and spleen were selected to profile the expression of the newly identified miRNAs (Figure 5).

The relative quantification results show that all novel miRNAs are present in different tissues of channel catfish. Most of the miRNAs are highly expressed in brain, heart, muscle and liver. Except for ipu-miR-7556, all novel miRNAs exhibited lower levels of expression in the skin and intestines. Some newly identified miRNAs, such as ipu-miR-129b, ipu-miR-7562 and ipu-miR-7553, demonstrate ubiquitous expression for all tissues examined. Ubiquitous expression of many miRNAs was reported in other species. The ubiquitous nature of these miRNAs indicates that they may be associated with fundamental functions, such as metabolism. Previous studies have suggested that tissue-specific miRNAs may be assessed as potential genetic markers for specific functional and diagnostic applications in aquaculture species [11]. In this study, ipu-miR-7147 and ipu-miR-203c were highly expressed in the heart, but relatively weakly expressed in the other tissues. Ipu-miR-7575 is predominantly expressed in the stomach. Tissue-specific differences in gene expression might represent polymorphism among individuals [44]. Therefore, these tissue-specific miRNAs may be associated with desired or undesired aquaculture production traits, such as growth and disease resistance, and may be assessed as potential genetic markers for specific functional studies. Moreover, hierarchical clustering analysis showed that some tissues exhibit similar patterns of miRNA expression. For example, similar expression profiles of many miRNAs were detected in head kidney and spleen which belong to the key tissues for fish immunity.

### Prediction of Potential miRNA Targets in Channel Catfish

The identification of miRNA targets is an important step to further understand the regulatory functions of miRNAs. Bioinformatic approaches have been used in many studies as an effective strategy to predict miRNA targets [11]–[14]. The conventional point of view is that miRNAs regulate target genes by binding to the 3′ UTRs of target mRNAs, and multiple binding sites for multiple miRNAs in 3′ UTRs can strongly enhance the degree of regulation. Recently, many studies have also demonstrated that a large number of miRNA binding sites reside in the 5′ UTR and the coding sequence of mRNAs [45]–[46].

Based on sequence complementarity between miRNAs and their mRNA targets, potential target sequences for the 45 novel miRNAs were identified by searching for antisense hits in the reference RNA sequences of the channel catfish. A total of 281 targets were predicted for the 45 novel miRNAs. Two or more target sequences were identified for all the novel miRNAs (Table S3). These potential target sequences are involved in immune regulation, transcriptional regulation, metabolism and many other biological functions. Some sequences are likely to be targeted by multiple miRNAs at multiple targeting sites. For example, Toll-like receptor 3 can be targeted by four miRNAs, including ipu-miR-101a-3p, ipu-miR-29a, ipu-miR-16c and ipu-miR-7553. The Toll-like receptor gene family is strongly associated with both the innate immune and adaptive immune systems in channel catfish [47]. The results indicate these four newly identified miRNAs are involved in regulating immunity of the channel catfish. It is, however, important for future studies to functionally validate the predictions of these miRNA targets.

### Conclusions

In this study, we have identified 237 conserved and 45 novel miRNAs in channel catfish using Solexa sequencing technology and bioinformatic analysis. The channel catfish is not only an economically important species in freshwater aquaculture around the world, but is also a model species for various biological studies in lower vertebrates. The channel catfish miRNAs identified and characterized in this study will provide new opportunities for future functional genome research in catfish and other fish species.

## Supporting Information

Table S1 **Primers used in this study for stem-loop real-time RT-PCR.**
(XLS)

Table S2 **Conserved miRNAs detected in channel catfish.**
(XLS)

Table S3 **Prediction of miRNA targets for the novel miRNAs in channel catfish.**
(DOC)
