# Supplementary material for: Identification and Characterization of MicroRNAs in Channel Catfish (Ictalurus punctatus) by Using Solexa Sequencing Technology
Source: PLoS One. 2013 Jan 16;8(1):e54174. doi: 10.1371/journal.pone.0054174 (PMC3546936; doi:10.1371/journal.pone.0054174)
Supplement: Table S3 — Prediction of miRNA targets for the novel miRNAs in channel catfish. (DOC) [file pone.0054174.s003.doc]

**Table S3. Prediction of miRNA targets for the novel miRNAs in channel catfish.**

| **miRNA** | **Gene Acc. No.** | **Gene Name** | **Energy**  (kcal/mol ) | **miRNA-target interaction** |
| --- | --- | --- | --- | --- |
| ipu-miR-24b | [NM_001200948.1](http://www.ncbi.nlm.nih.gov/nucleotide/318056275?report=genbank&log$=nucltop&blast_rank=19&RID=7W4UCS92014) | Ictalurus punctatus cellular repressor of E1A-stimulated genes 1 (creg1), mRNA | -24.7 | position 524  target 5'G U AA UG AG G 3'  GUUC UUGCU GA GC UGAGCCA  CAAG GACGA CU UG ACUCGGU  miRNA 3' 5' |
|  | NM_001200578.1 | Ictalurus punctatus tetraspanin-31 (tsn31), mRNA | -28.6 | position 6  target 5'G AGGU A 3'  GUUUUUGC UGAACUGAGC  CAAGGACG ACUUGACUCG  miRNA 3' GU 5' |
|  | NM_001200214.1 | Ictalurus punctatus growth hormone receptor (LOC100304685), mRNA | -27.6 | position 476  target 5' A ACG G 3'  CCUG GAGCUGGGC  GGAC CUUGACUCG  miRNA 3'CAA GA GU 5' |
|  | NM_001200170.1 | Ictalurus punctatus Oct1 transcription factor (oct1), mRNA | -24.9 | position 957  target 5'C A A 3'  UUUCUG GAGC GAGCCA  AAGGAC CUUG CUCGGU  miRNA 3' C GA A 5' |
|  | NM_001200314.1 | Ictalurus punctatus cytochrome P4501B (cyp1b), mRNA | -26.9 | position 1390  target5' A GCG AU U 3'  CC GC GACUGGGCCA  GG CG UUGACUCGGU  miRNA 3' CAA A AC 5' |
|  | NM_001200171.1 | Ictalurus punctatus Oct2 transcription factor (oct2), mRNA | -25.3 | position 1413  target 5' A CAAAU C 3'  UUUUGCUG CUGAGCU  AGGACGAC GACUCGG  miRNA 3' CA UU U 5' |
|  | NM_001200883.1 | Ictalurus punctatus proteasome (prosome, macropain) 26S subunit, non-ATPase, 8 (psmd8), mRNA | -30.0 | position 527  target 5' A U 3'  UUCCUGCUGA GAGCUA  AAGGACGACU CUCGGU  miRNA 3' C UGA 5' |
|  | NM_001200066.1 | Ictalurus punctatus NLR family, CARD domain containing 5 (nlrc5), mRNA | -29.4 | position 4793  target 5' A GCA U G 3'  GUUCU GCUG ACUGGGUCA  CAAGG CGAC UGACUCGGU  miRNA 3' A U 5' |
|  | NM_001200211.1 | Ictalurus punctatus Mx2 protein (mx2), mRNA | -24.4 | position 2088  target 5' C ACG U U 3'  UUUCUGU G GCUGAGUC  AAGGACG C UGACUCGG  miRNA 3' C A U U 5' |
| ipu-miR-7547 | NM_001200933.1 | Ictalurus punctatus endothelial differentiation-related factor 1 (edf1), mRNA | -28.3 | position 515  target 5' U UUACAGUC CAC G 3'  GGCC CGUUUC AUGCUGCU  CCGG GCGAAG UGCGGCGA  miRNA 3' UA AC 5' |
|  | NM_001200066.1 | Ictalurus punctatus NLR family, CARD domain containing 5 (nlrc5), mRNA | -29.5 | position 3210  target 5' U U A U U A G 3'  GG CC AUUGCUU CUGA UGCC GC  CC GG UAGCGAA GACU GCGG CG  miRNA 3' A 5' |
|  | NM_001200252.1 | Ictalurus punctatus cathspsin H (LOC100305012), mRNA | -28.9 | position 455  target 5' A GGG G A G 3'  UCA UGCUU UGGC GCUGCU  GGU GCGAA ACUG CGGCGA  miRNA 3' CC A G 5' |
|  | NM_001200104.1 | Ictalurus punctatus ribosomal protein L3 (LOC100304518), mRNA | -29.1 | position 325  target 5' A A A GAUGAGUG A U 3'  GC CAUC GU C GACGCCGCU  CG GUAG CG G CUGCGGCGA  miRNA 3' C AA A 5' |
|  | NM_001200074.1 | Ictalurus punctatus estrogen receptor type alpha (er), mRNA | -29.4 | position 4244  target 5' C A UGAA A G 3'  GG CCGUC GCUUCUGGC CGU  CC GGUAG CGAAGACUG GCG  miRNA 3' CG A 5' |
|  | NM_001200314.1 | Ictalurus punctatus cytochrome P4501B (cyp1b), mRNA | -31.1 | position 271  target 5' A U GCA A 3'  CAUU GCUUU GACGCCGC  GUAG CGAAG CUGCGGCG  miRNA 3' CCG A A 5' |
|  | NM_001200212.1 | Ictalurus punctatus hypoxia induced factor-like factor (LOC100304653), mRNA | -31.2 | position 200  target 5' U CAU GCCC UC A 3'  GCC UCGUU CUG ACGCCGC  CGG AGCGA GAC UGCGGCG  miRNA 3'C U A A 5' |
| Ipu-miR-101a | NM_001200068.1 | Ictalurus punctatus Toll-like receptor 3 (LOC100303712), mRNA | -27.4 | position 1720  target 5' U GG C 3'  GG GUC CAGUGCUGAU  UC UAG GUCACGACUA  miRNA 3' AG AA U C 5' |
|  | NM_001201023.1 | Ictalurus punctatus phosphoribosyltransferase domain-containing protein 1 (prdc1), mRNA | -21.2 | position 329  target 5'U GGAACG UCA A 3'  GGU CAU GGUGCUGA  UCA GUG UCACGACU  miRNA 3' AG AUA AC 5' |
|  | NM_001200066.1 | Ictalurus punctatus NLR family, CARD domain containing 5 (nlrc5), mRNA | -28.3 | position 1082  target 5' G G C 3'  GGU CACAGUGCUGGU  UCA GUGUCACGACUA  miRNA 3' AG AUA C 5' |
|  | NM_001200234.1 | Ictalurus punctatus HIF 2 alpha (LOC100304992), mRNA | -28.0 | position 4071  target 5' C AGAUGUGG G U 3'  UCAGU AUCA AGUGCUGAU  AGUCA UAGU UCACGACUA  miRNA 3' A G C 5' |
| ipu-miR-7147 | NM_001201288.1 | Ictalurus punctatus lactate dehydrogenase A4 (ldha), mRNA | -29.5 | position 252  target 5' A GGACCUG G 3'  GGCUAU CAGCAUGGUA  CCGAUG GUCGUACCAU  miRNA 3' UGA GU 5' |
|  | NM_001201133.1 | Ictalurus punctatus cysteine and histidine-rich domain-containing protein 1 (chrd1), mRNA | -25.9 | position 841  target 5' C A UA A A 3'  CUG GCU GCCA CGUGG UGCG  GAC CGA UGGU GUACC AUGU  miRNA 3' U C 5' |
| ipu-miR-29a | NM_001200884.1 | Ictalurus punctatus pancreatic triacylglycerol lipase (lipp), mRNA | -27.5 | position 213  target 5' G AUUGCUAC A 3'  AACACUG GAGGAAAUCAGU  UUGUGGU CUCCUUUAGUCA  miRNA 3' 5' |
|  | NM_001200812.1 | Ictalurus punctatus leptin receptor gene-related protein (obrg), mRNA | -22.9 | position 461  target 5' U AGGUUG A 3'  GCC AGAGGAAAUCA  UGG UCUCCUUUAGU  miRNA 3' UUG CA 5' |
|  | NM_001200540.1 | Ictalurus punctatus crystallin, lambda 1 (cryl1), mRNA | -25.5 | position 282  target 5' A U 3'  GGCACUAGAGGGAG CA  UUGUGGUCUCCUUU GU  miRNA 3' A CA 5' |
|  | NM_001200067.1 | Ictalurus punctatus nucleotide-binding oligomerization domain containing 1 (nod1), mRNA | -24.3 | position 843  target 5' A A U C 3'  GACAC CAGA GGAAAUC  UUGUG GUCU CCUUUAG  miRNA 3' UCA 5' |
|  | NM_001200275.1 | Ictalurus punctatus leukocyte DNA binding receptor (LOC100305038), mRNA | -23.3 | position 529  target 5' G UG G A 3'  U CAGA GGAGAUCA  G GUCU CCUUUAGU  miRNA 3' UU UG CA 5' |
|  | NM_001200068.1 | Ictalurus punctatus Toll-like receptor 3 (LOC100303712), mRNA | -24.3 | position 208  target 5' G UU U C C 3'  GAC GCC GAGGA AUCAG  UUG UGG CUCCU UAGUC  miRNA 3' U U A 5' |
|  | NM_001200230.1 | Ictalurus punctatus toll-like receptor 20a (tlr-20a), mRNA | -22.7 | position 1363  target 5' C U G A 3'  AGCACU GAGGAGGU G  UUGUGG CUCCUUUA U  miRNA 3' U G CA 5' |
|  | NM_001200298.1 | Ictalurus punctatus E2A-1 transcription factor (LOC100305065), mRNA | -28.1 | position 1968  target 5' G CU C 3'  AGCACCAGAGGG UCA  UUGUGGUCUCCU AGU  miRNA 3' UU CA 5' |
|  | NM_001200273.1 | Ictalurus punctatus heat shock protein 70 (LOC100305036), mRNA | -24.0 | position 777  target 5' A A A C G 3'  AGCAC AG AGGA AUCAGU  UUGUG UC UCCU UAGUCA  miRNA 3' G U 5' |
| ipu-miR-16c | NM_001201269.1 | Ictalurus punctatus ADP-ribosylation factor 5 (arf5), mRNA | -32.2 | position 482  target 5' G GGAC U 3'  AG ACCGUGCUGCUGG  UC UGGCACGACGACC  miRNA 3' G AUAAC 5' |
|  | NM_001201313.1 | Ictalurus punctatus CASP2 and RIPK1 domain containing adaptor with death domain (cradd), mRNA | -33.4 | position 524  target 5' G GGA G A 3'  AGUG GAU GUGCUGCUGG  UCAU CUG CACGACGACC  miRNA 3' G AA G 5' |
|  | NM_001200371.1 | Ictalurus punctatus neural precursor cell expressed, developmentally down-regulated 8 (nedd8), mRNA | -26.3 | position 460  target 5' A GC C 3'  CAGUG GACU CUGCUGG  GUCAU CUGG GACGACC  miRNA 3' AA CAC 5' |
|  | NM_001200083.1 | Ictalurus punctatus estrogen receptor type beta (LOC100304489), mRNA | -29.6 | position 1640  target 5' U A 3'  GUA UGACC UGUUGCUGG  CAU ACUGG ACGACGACC  miRNA 3' GU A C 5' |
|  | NM_001257112.1 | Ictalurus punctatus protein arginine methyltransferase (prmt4), mRNA | -26.7 | position 1135  target 5' U A G C 3'  GUG UGACCGUGU GCU  CAU ACUGGCACG CGA  miRNA 3' GU A A CC 5' |
|  | NM_001200068.1 | Ictalurus punctatus Toll-like receptor 3 (LOC100303712), mRNA | -27.8 | position 699  target 5' A G U 3'  GGUG GACCGU GCUGCU  UCAU CUGGCA CGACGA  miRNA 3' G AA CC 5' |
|  | NM_001200067.1 | Ictalurus punctatus nucleotide-binding oligomerization domain containing 1 (nod1), mRNA | -27.2 | position 1481  target 5' U A A 3'  GGUGU CUGUGC GCUGG  UCAUA GGCACG CGACC  miRNA 3' G ACU A 5' |
|  | NM_001200192.1 | Ictalurus punctatus thyroid stimulating hormone receptor (LOC100304626), mRNA | -24.7 | position 912  target 5' U GU A U 3'  UAGUG CC GUGCUGCU  GUCAU GG CACGACGA  miRNA 3' AACU CC 5' |
|  | NM_001200273.1 | Ictalurus punctatus heat shock protein 70 (LOC100305036), mRNA | -24.9 | position 42  target 5' A G A C 3'  AG GACC GCUGUUGG  UC CUGG CGACGACC  miRNA 3' G AUAA CA 5' |
| ipu-miR-199b | NM_001201067.1 | Ictalurus punctatus cathepsin L (catl), mRNA | -21.5 | position 862  target 5' C CUG U 3'  AUGGU GUC GC UGUUGGUUA  UGUCA CAG CG GUAACCAAU  miRNA 3' U A U 5' |
|  | NM_001200898.1 | Ictalurus punctatus arylamine n-acetyltransferase pineal gland isozyme nat-10 (ary1), mRNA | -21.3 | position 768  target 5' U UG G 3'  CAG AG CU C AUUGGUUG  GUC UC GA G UAACCAAU  miRNA 3' U A A C UG 5' |
|  | NM_001200644.1 | Ictalurus punctatus acyl-protein thioesterase 2 (lypa2), mRNA | -23.6 | position 461  target 5' A GU GCUCUAA C 3'  G GUAGUC GC UGUUGGUUA  U CAUCAG CG GUAACCAAU  miRNA 3' GU A U 5' |
|  | NM_001200899.1 | Ictalurus punctatus sulfotransferase family cytosolic 2b member 1 (st2b1), mRNA | -24.2 | position 1105  target 5' C CAACCAC U U 3'  ACAG UUUGCAC UUGGUUA  UGUC AGACGUG AACCAAU  miRNA 3' AUC U 5' |
| ipu-miR-7548 | NM_001200221.1 | Ictalurus punctatus FcRI (LOC100304698), mRNA | -28.1 | position 139  target 5' A GGGAA G 3'  GUUU AGCCGCGGCU  CGAG UCGGCGCCGA  miRNA 3' GAUG 5' |
|  | NM_001201043.1 | Ictalurus punctatus DNA-directed RNA polymerase II subunit rpb4 (rpb4), mRNA | -29.7 | position 500  target 5' G A A 3'  UCCUG AGCCGCGG  AGGAU UCGGCGCC  miRNA 3'CG G GA 5' |
|  | NM_001201021.1 | Ictalurus punctatus ependymin-1 (epd1), mRNA | -32.3 | position 73  target 5' U C A C 3'  CUCCU CAGCCGCG GC  GAGGA GUCGGCGC CG  miRNA 3' C U A 5' |
|  | NM_001200815.1 | Ictalurus punctatus transmembrane protein 41a-a (t41aa), mRNA | -30.1 | position 78  target 5' C C 3'  CUCCUGUA CCGCGGCU  GAGGAUGU GGCGCCGA  miRNA 3'C C 5' |
|  | NM_001200204.1 | Ictalurus punctatus CXCL14 (LOC100304643), mRNA | -27.3 | position 213  target 5' C G U 3'  GCU C ACAGCCGCG  CGA G UGUCGGCGC  miRNA 3' G A CGA 5' |
|  | NM_001201113.1 | Ictalurus punctatus transmembrane protein 144 (tm144), mRNA | -32.2 | position 1146  target 5' A G UGUUU G 3'  CU CCUGC GCCGCGGC  GA GGAUG CGGCGCCG  miRNA 3'C U A 5' |
| ipu-miR-203c | NM_001201127.1 | Ictalurus punctatus transmembrane protein 82 (tmm82), mRNA | -26.9 | position 380  target 5' U U 3'  UAGUGGUUCUUG AGUUC  GUCACCAAGAAU UCAAG  miRNA 3'C UG UU 5' |
|  | NM_001200742.1 | Ictalurus punctatus isocitrate dehydrogenase 3 (NAD+) alpha , nuclear gene encoding mitochondrial protein, mRNA | -21.0 | position 593  target 5' C UAAC C 3'  CAGUG AGCAGUUCA  GUCAC UUGUCAAGU  miRNA 3' C CAAGAA U 5' |
|  | NM_001200804.1 | Ictalurus punctatus ADP-ribosylation factor-like 2 (arl2), mRNA | -23.4 | position 375  target 5' G G G A 3'  GGUGGU GACAGU CAG  UCACCA UUGUCA GUU  miRNA 3' CG AGAA A 5' |
| ipu-miR-551 | NM_001201093.1 | Ictalurus punctatus lysm and putative peptidoglycan-binding domain-containing protein 3 (lysm3), mRNA | -23.4 | position 718  target 5' C GG U A G G 3'  GGACU GG AUG GGU GU  UUUGG CC UAC CCA CG  miRNA 3' UC UU G 5' |
|  | NM_001200812.1 | Ictalurus punctatus leptin receptor gene-related protein (obrg), mRNA | -25.7 | position 444  target 5' C UG A G C 3'  G GCCAA GA GGGUUGC  C UGGUU CU CCCAGCG  miRNA 3' U UU C A 5' |
| ipu-miR-7549 | NM_001200355.1 | Ictalurus punctatus asparaginase homolog (S. cerevisiae) (aspg), mRNA | -32.8 | position 869  target 5' C C AAA CC A 3'  GCCGCCGC UGC ACCG AUC  CGGCGGCG ACG UGGC UAG  miRNA 3' 5' |
|  | NM_001201107.1 | Ictalurus punctatus translation initiation factor eif-2b subunit alpha (ei2ba), mRNA | -29.1 | position 541  target 5' C GA U G U 3'  GUCGCUG GCA CC GAUC  CGGCGGC CGU GG CUAG  miRNA 3' GA 5' |
|  | NM_001200455.1 | Ictalurus punctatus glyoxylate reductase/hydroxypyruvate reductase (grhpr), mRNA | -26.9 | position 918  target 5' U C U 3'  CCGCU CUGCGCC  GGCGG GACGUGG  miRNA 3'C C CUAG 5' |
|  | NM_001201105.1 | Ictalurus puncta tus canopy1 (cnpy1), mRNA | -33.3 | position 626  target 5' A A 3'  CCGCCGCUG ACCGA  GGCGGCGAC UGGCU  miRNA 3' C G AG 5' |
|  | NM_001200663.1 | Ictalurus punctatus ADP-ribosylarginine hydrolase (adprh), mRNA | -26.4 | position 421  target 5' U AUGAGG UAUG U 3'  GCUGCC GC UGCAUUGGUC  CGGCGG CG ACGUGGCUAG  miRNA 3' 5' |
| ipu-miR-129b | NM_001200590.1 | Ictalurus punctatus steroidogenic acute regulatory protein (star), nuclear gene encoding mitochondrial protein, mRNA | -31.8 | position 650  target 5' U UGUC A AAC C 3'  GGCCGGGA G CCC AUCCCAG  UCGGUCCU C GGG UGGGGUU  miRNA 3' U AA U 5' |
|  | NM_001200067.1 | Ictalurus punctatus nucleotide-binding oligomerization domain containing 1 (nod1), mRNA | -33.1 | position 3348  target 5' U U CCCU AG U 3'  GCCA GA GCCC GCCCCA  CGGU CU CGGG UGGGGU  miRNA 3' U C U AA UU 5' |
|  | NM_001200260.1 | Ictalurus punctatus annexin A11 (LOC100305021), mRNA | -35.5 | position 260  target 5' C C UUAC GGCA G 3'  AGCC GGAGG CCC UGCCCCAAG  UCGG CCUUC GGG AUGGGGUUU  miRNA 3' U A 5' |
|  | NM_001200278.1 | Ictalurus punctatus myeloid differentiation primary response gene (88) (myd88), mRNA | -33.0 | position 889  target 5' A CAUGAA C 3'  AGCC GAAGCCCUU UCCCAG  UCGG CUUCGGGAA GGGGUU  miRNA 3' UC U U 5' |
|  | NM_001200298.1 | Ictalurus punctatus E2A-1 transcription factor (LOC100305065), mRNA | -34.3 | position 1554  target 5' C UU G 3'  GCCAG GGCUC GCCCCAGG  CGGUC UCGGG UGGGGUUU  miRNA 3' U CU AA 5' |
|  | NM_001200104.1 | Ictalurus punctatus ribosomal protein L3 (LOC100304518), mRNA | -29.1 | position 71  target 5' C CCGUGGUAAGGU AG G 3'  GCCAG GAAG CUU CCCCAAA  CGGUC CUUC GAA GGGGUUU  miRNA 3' U GG U 5' |
| ipu-miR-7550 | NM_001201001.1 | Ictalurus punctatus akirin-1 (akir1), mRNA | -24.1 | position 216  target 5' A C AGC G 3'  GUCC CGA GCCGGA  CAGG GCU CGGCCU  miRNA 3' AC AA A 5' |
|  | NM_001200973.1 | Ictalurus punctatus four and a half LIM domains 3 (fhl3), mRNA | -27.9 | position 851  target 5' C G U 3'  UGGUC GAGCCGGAU  ACCAG CUCGGCCUA  miRNA 3' GAAG 5' |
|  | NM_001200301.1 | Ictalurus punctatus hypoxia induced factor 1 alpha (LOC100305068), mRNA | -28.0 | position 2073  target 5' A G G 3'  GG CU UCGGGCUGGAU  CC GG AGCUCGGCCUA  miRNA 3' A A A 5' |
|  | NM_001200074.1 | Ictalurus punctatus estrogen receptor type alpha (er), mRNA | -27.3 | position 4227  target 5' A AAAG C C 3'  UGGUCCU C AGUCGGA  ACCAGGA G UCGGCCU  miRNA 3' A C A 5' |
| ipu-miR-3618 | NM_001200365.1 | Ictalurus punctatus mitochondrial GTPase 1 (mtg1), nuclear gene encoding mitochondrial protein, mRNA | -23.2 | position 673  target 5' A C GG G 3'  G CUCAA GUUGGAGGU  C GAGUU UAACCUUUA  miRNA 3' UGA A AA G 5' |
|  | NM_001200965.1 | Ictalurus punctatus mitochondrial creatine kinase s-type (kcrs), nuclear gene encoding mitochondrial protein, mRNA | -20.7 | target 5' U G C AAA U 3'  UUGUCU GA UG GGAGAUC  GACAGA UU AU CCUUUAG  miRNA 3' U G A AA 5' |
|  | NM_001201155.1 | Ictalurus punctatus cholesterol 25-hydroxylase (ch25h), mRNA | -23.5 | position 79  target 5' A CUCCA U 3'  UGUCUCAG GGGAAUC  ACAGAGUU CCUUUAG  miRNA 3' UG AAUAA 5' |
|  | NM_001200607.1 | Ictalurus punctatus trafficking protein particle complex subunit 4 (tppc4), mRNA | -25.2 | position 1113  target 5' G GUCA U 3'  UUGUCUCAAU GUUGGAG  GACAGAGUUA UAACCUU  miRNA 3' U A UAG 5' |
|  | NM_001200326.1 | Ictalurus punctatus annexin A6 (LOC100335034), mRNA | -21.1 | position 1040  target 5' U AAG U GG G 3'  GCUG CUC GU UGGAGAU  UGAC GAG UA ACCUUUA  miRNA 3' A U AUA G 5' |
|  | NM_001200066.1 | Ictalurus punctatus NLR family, CARD domain containing 5 (nlrc5), mRNA | -25.6 | position 5314  target 5' U GAAUA A 3'  CUGUUUCAAUUA UGGAGGU  GACAGAGUUAAU ACCUUUA  miRNA 3' U A G 5' |
|  | NM_001200069.1 | Ictalurus punctatus a disintegrin and metalloproteinase domain 8 (adam8), mRNA | -23.3 | position 1691  target 5' U G G 3'  ACUG CUCAG UGGAAAU  UGAC GAGUU ACCUUUA  miRNA 3' A AAUA G 5' |
|  | NM_001200100.1 | Ictalurus punctatus natural resistance-associated macrophage protein large transcript (LOC100304514), mRNA | -20.9 | position 2934  target 5' U AACGUUAUAUUUAA G 3'  CUGUCU AAU UGUUGGAAAU  GACAGA UUA AUAACCUUUA  miRNA 3' U G G 5' |
|  | NM_001200165.1 | Ictalurus punctatus cyclic nucleotide-gated channel modulatory subunit (cnga4), mRNA | -21.2 | position 2038  target 5' G GACA G 3'  UGUCUUAA UGGAGA  ACAGAGUU ACCUUU  miRNA 3' UG AAUA AG 5' |
| ipu-miR-7551 | NM_001200308.1 | Ictalurus punctatus insulin-like growth factor binding protein 1 (LOC100305121), mRNA | -35.0 | position 1663  target 5' U U C 3'  CCA GGACUUAGGCCCC  GGU CCUGAGUCCGGGG  miRNA 3' CUU 5' |
|  | NM_001200624.1 | Ictalurus punctatus nedd4 family-interacting protein 1-like (nfi1l), mRNA | -27.8 | position 188  target 5' A C GAG U 3'  GGAGGGG CUCAGGU CUC  UCUUCCU GAGUCCG GGG  miRNA 3' GG 5' |
|  | NM_001200553.1 | Ictalurus punctatus methylmalonic aciduria and homocystinuria type c-like protein (mmac), mRNA | -28.5 | position 113  target 5' G UU UUU A 3'  CAG G CUCAGGCCC  GUC C GAGUCCGGG  miRNA 3' G UU CU G 5' |
|  | NM_001200180.1 | Ictalurus punctatus calnexin (LOC100304606), mRNA | -29.2 | position 1409  target 5' A G G 3'  GGAAGG CU GGGCCU  UCUUCC GA UCCGGG  miRNA 3' GG U G G 5' |
| ipu-miR-7552 | NM_001200225.1 | Ictalurus punctatus TIR-containing adaptor molecule (LOC100304977), mRNA | -23.8 | position 915  target 5' A C GAGG A G 3'  AGCCAG CAG AAG GGACAU  UUGGUU GUU UUC CCUGUA  miRNA 3' U AA A 5' |
|  | NM_001200325.1 | Ictalurus punctatus annexin A1 (LOC100335033), mRNA | -25.3 | position 864  target 5' C U U 3'  AACC GGCAAUUAAGGG C  UUGG UUGUUAAUUCCC G  miRNA 3' U U UAA 5' |
|  | NM_001200318.1 | Ictalurus punctatus E-box binding protein 1 (eb1), mRNA | -21.9 | position 1525  target 5' C UGACAUCC UGCU G 3'  CCAG ACAGUU GGGACA  GGUU UGUUAA CCCUGU  miRNA 3' UU UU AA 5' |
|  | NM_001201114.1 | Ictalurus punctatus serine/threonine-protein phosphatase dullard-a (dulda), mRNA | -20.4 | position 816  target 5' U CU C A 3'  GGCU GCAGU AG GGACA  UUGG UGUUA UC CCUGU  miRNA 3' UU AU AA 5' |
|  | NM_001200894.1 | Ictalurus punctatus upf0739 protein c1orf74-like protein (ca074), mRNA | -21.5 | position 161  target 5' U UU UA A A 3'  AGCC GC UAGGG GACA  UUGG UG AUUCC CUGU  miRNA 3' UU UUA AA 5' |
|  | NM_001200662.1 | Ictalurus punctatus RNA (guanine-9-)-methyltransferase domain-containing protein 3 (rg9d3), mRNA | -21.4 | position 120  target 5' U UC U A 3'  GGCCA UA GGGACAUU  UUGGU GU CCCUGUAA  miRNA 3' UU UAAUU 5' |
|  | NM_001200065.1 | Ictalurus punctatus toll-like receptor 21 (tlr-21), mRNA | -22.2 | position 2123  target 5' A AUU AACAU C 3'  GAC AACAGUUGAG GGGCAU  UUG UUGUUAAUUC CCUGUA  miRNA 3' GU A 5' |
|  | NM_001200214.1 | Ictalurus punctatus growth hormone receptor (LOC100304685), mRNA | -24.7 | position 1907  target 5' C G UCC U 3'  AA CAAACA UGGGGGAUAUU  UU GUUUGU AUUCCCUGUAA  miRNA 3' G UA 5' |
|  | NM_001200199.1 | Ictalurus punctatus IGF2 (LOC100304637), mRNA | -23.7 | position 1250  target 5' C UU CU A C 3'  ACCG CA AU AAGGGACAU  UGGU GU UA UUCCCUGUA  miRNA 3' U UU A A 5' |
| ipu-miR-7553 | NM_001201233.1 | Ictalurus punctatus nicotinamide mononucleotide adenylyltransferase 1 (nmna1), mRNA | -22.5 | position 209  target 5' A AAA AUCAUCUCUCC C U 3'  GU GGGU UGUUGGUGAUG C  CA CCCA GCGAUUACUGC G  miRNA 3' C G A U 5' |
|  | NM_001200068.1 | Ictalurus punctatus Toll-like receptor 3 (LOC100303712), mRNA | -27.1 | position 2854  target 5' A UUA A U 3'  GGGUUGCUG AUGAU GUC  CCCAGCGAU UACUG CAG  miRNA 3' CCAG U 5' |
|  | NM_001200620.1 | Ictalurus punctatus B-cell translocation gene 1 (btg1), mRNA | -29.2 | position 7  target 5' C C C GCA U 3'  GUC GG CGU GUGACGUCA  CAG CC GCG UACUGCAGU  miRNA 3' C C A AU 5' |
|  | NM_001200082.1 | Ictalurus punctatus EB1 (LOC100304488), mRNA | -26.6 | position 1242  target 5' A G U U 3'  UGGGU GC AUG ACGUCA  GCCCA CG UAC UGCAGU  miRNA 3' CCA G AU 5' |
| ipu-miR-7554 | NM_001200468.1 | Ictalurus punctatus upf0533 protein c5orf44-like protein (ce044), mRNA | -22.7 | position 4  target 5' G CUCU G 3'  UCAG UAGACAAAAUG  AGUC AUCUGUUUUAC  miRNA 3' AA U A 5' |
|  | NM_001200737.1 | Ictalurus punctatus colipase (col), mRNA | -23.4 | position 396  target 5' A U 3'  UUUCAGAUAGGCAA  AAAGUCUAUCUGUU  miRNA 3' UUACA 5' |
|  | NM_001200662.1 | Ictalurus punctatus RNA (guanine-9-)-methyltransferase domain-containing protein 3 (rg9d3), mRNA | -20.5 | position 145  target 5' A C C 3'  UAGA AGACAAAAUG  GUCU UCUGUUUUAC  miRNA 3' AAA A A 5' |
|  | NM_001200179.1 | Ictalurus punctatus granzyme (LOC100304605), mRNA | -21.5 | position 452  target 5' C CAG G 3'  CAG GGACAAAAUGU  GUC UCUGUUUUACA  miRNA 3' AAA UA 5' |
|  | NM_001200203.1 | Ictalurus punctatus CXCL12 (LOC100304642), mRNA | -22.1 | position 652  target 5' G CU A 3'  UUAGGUAGAC GAAUGU  AGUCUAUCUG UUUACA  miRNA 3' AA U 5' |
|  | NM_001201275.1 | Ictalurus punctatus G protein pathway suppressor 2 (gps2), mRNA | -20.0 | position 286  target 5' A AG A 3'  AGA UGGACAAAAUG  UCU AUCUGUUUUAC  miRNA 3' AAAG A 5' |
|  | NM_001200065.1 | Ictalurus punctatus toll-like receptor 21 (tlr-21), mRNA | -21.3 | position 1902  target 5' G U 3'  UUAGAUGGACAAA  AGUCUAUCUGUUU  miRNA 3' AA UACA 5' |
|  | NM_001200228.1 | Ictalurus punctatus matrix metalloproteinase 9 (mmp9), mRNA | -21.0 | position 1819  target 5' G C A 3'  UUCA UGGACAGGAUGU  AAGU AUCUGUUUUACA  miRNA 3' A CU 5' |
| ipu-miR-7555 | NM_001200310.1 | Ictalurus punctatus mammal-like melanopsin 2 (opn4m2), mRNA | -38.8 | position 1457  target 5' C A C 3'  CCUGGGUGUCCGG UUGG  GGACCCACAGGCC GACC  miRNA 3' CA C UC 5' |
|  | NM_001201106.1 | Ictalurus punctatus TOB1 protein (tob1), mRNA | -28.6 | position 328  target 5' G CUGUAUUCAC A 3'  UCUGGGU UCCG GUUGGGG  GGACCCA AGGC CGACCUC  miRNA 3' CA C C 5' |
|  | NM_001200263.1 | Ictalurus punctatus vertebrate ancient long opsin (LOC100305024), mRNA | -40.2 | position 420  target 5' G C G U G 3'  GUCCUGGG GU CG GCUGGAG  CAGGACCC CA GC CGACCUC  miRNA 3' A G C 5' |
|  | NM_001201067.1 | Ictalurus punctatus cathepsin L (catl), mRNA | -29.6 | position 860  target 5' A AU U UU G 3'  UC GGUGUCC GGCUG G  AG CCACAGG CCGAC C  miRNA 3' C GAC C CU 5' |
|  | NM_001201291.1 | Ictalurus punctatus charged multivesicular body protein 1a (chm1a), mRNA | -28.9 | position 473  target 5' A C AGGA AG G 3'  UUG GG G UGGGCUGGAG  GAC CC C GCCCGACCUC  miRNA 3' CAG A AG 5' |
|  | NM_001200679.1 | Ictalurus punctatus down syndrome critical region protein 3-like protein (dscr3), mRNA | -35.6 | position 116  target 5' A GAG U U 3'  GGG GUCC GGCUGGAG  CCC CAGG CCGACCUC  miRNA 3' CAGGA A C 5' |
|  | NM_001200234.1 | Ictalurus punctatus HIF 2 alpha (LOC100304992), mRNA | -28.4 | position 1956  target 5' A A A AC A 3'  CUG GUG UCUGG CUGGAG  GAC CAC AGGCC GACCUC  miRNA 3' CAG C C 5' |
|  | NM_001200301.1 | Ictalurus punctatus hypoxia induced factor 1 alpha (LOC100305068), mRNA | -31.8 | position 2067  target 5' U U A C U 3'  GUC C GG UGU CGGGCUGGA  CAG G CC ACA GCCCGACCU  miRNA 3' A C G C 5' |
|  | NM_001200074.1 | Ictalurus punctatus estrogen receptor type alpha (er), mRNA | -33.4 | position 61  target 5' U U G U A 3'  UCCUG GUG UCUGG GUUGGA  AGGAC CAC AGGCC CGACCU  miRNA 3' C C C 5' |
| ipu-miR-7556 | NM_001200234.1 | Ictalurus punctatus HIF 2 alpha (LOC100304992), mRNA | -21.0 | position 3366  target 5' C A A 3'  UG UGU UUAAGUUGCUG  GC ACA AGUUCAAUGAC  miRNA 3'U A GA AU 5' |
|  | NM_001200083.1 | Ictalurus punctatus estrogen receptor type beta (LOC100304489), mRNA | -25.1 | position 1986  target 5' G GAAG U C U 3'  GU GU CUUCA AGUUACUGUA  CA CA GAAGU UCAAUGACAU  miRNA 3'UG A 5' |
|  | NM_001200925.1 | Ictalurus punctatus brain protein 44-like protein (br44l), mRNA | -21.1 | position 710  target 5' A A GUACA C A 3'  UGU UG CUUCAAG UACUG  GCA AC GAAGUUC AUGAC  miRNA 3'U A A AU 5' |
|  | NM_001200066.1 | Ictalurus punctatus NLR family, CARD domain containing 5 (nlrc5), mRNA | -22.2 | position 4880  target 5' G GU A 3'  GUUGU UCAAGU CUGUG  CAACA AGUUCA GACAU  miRNA 3'UG GA AU 5' |
| ipu-miR-7557 | NM_001257113.1 | Ictalurus punctatus protein arginine methyltransferase (co-prmt), mRNA | -29.7 | position 2  target 5' U C UG A U 3'  GGG AGCAG AGGA GC AAGC  CCC UUGUC UCUU CG UUCG  miRNA 3' U UG G U 5' |
|  | NM_001200067.1 | Ictalurus punctatus nucleotide-binding oligomerization domain containing 1 (nod1), mRNA | -30.0 | position 218  target 5' C U CU C U 3'  AGGGAAC GAU GAUGU AGCA  UCCCUUG CUG UUGCG UCGU  miRNA 3' U UC U 5' |
| ipu-miR-7558a | NM_001201004.1 | Ictalurus punctatus osteoclast stimulating factor 1 (ostf1), mRNA | -32.0 | position 11  target 5' C C C U 3'  GG UGCUCCCAAUCUC GUU  CC ACGAGGGUUAGAG CGG  miRNA 3' U CUC U 5' |
|  | NM_001201248.1 | Ictalurus punctatus cell division cycle 42 (cdc42), mRNA | -33.9 | position 543  target 5' C U GC C A 3'  G GGAGUGCUCC UCUCA GC  C CCUCACGAGG AGAGU CG  miRNA 3' U GUU G 5' |
|  | NM_001200389.1 | Ictalurus punctatus fam49a (fa49a), mRNA | -29.2 | position 23  target 5' U A A 3'  GGGGG AAUCUCAGCC  CCCUC UUAGAGUCGG  miRNA 3' U ACGAGGG 5' |
|  | NM_001200171.1 | Ictalurus punctatus Oct2 transcription factor (oct2), mRNA | -32.2 | position 769  target 5' U C U 3'  GAG GC CUCAAUCUCAGCU  CUC CG GGGUUAGAGUCGG  miRNA 3' UCC A A 5' |
|  | NM_001200313.1 | Ictalurus punctatus steroidogenic cytochrome P450 17-hydroxylase/lyase (cyp17), mRNA | -42.0 | position 1126  target 5' C A G A 3'  AGGG AGUGCUCC AAUC CGGCC  UCCC UCACGAGG UUAG GUCGG  miRNA 3' G A 5' |
|  | NM_001257110.1 | Ictalurus punctatus cathepsin D1 (LOC100862736), mRNA | -36.9 | position 432  target 5' C UGGCU A 3'  GGGAGU CUUUC AUCUCAGCC  CCCUCA GAGGG UAGAGUCGG  miRNA 3' U C U 5' |
|  | NM_001200165.1.1 | Ictalurus punctatus cyclic nucleotide-gated channel modulatory subunit (cnga4), mRNA | -30.1 | position 1713  target 5' A U C A G U 3'  AG GGAG GC UC AGUC CAGCC  UC CCUC CG GG UUAG GUCGG  miRNA 3' A A G A 5' |
| ipu-miR-7559 | NM_001200298.1 | Ictalurus punctatus E2A-1 transcription factor (LOC100305065), mRNA | -33.8 | position 276  target 5' C U AACAAAAAAAGGGA G 3'  AGUUG AGC AGUCGUGUGGCA  UCGAC UCG UCAGCACACCGU  miRNA 3' AC 5' |
|  | NM_001200066.1 | Ictalurus punctatus NLR family, CARD domain containing 5 (nlrc5), mRNA | -34.9 | position 764  target 5' A UUCUGUGACU U U 3'  AGCUG GC GUCGUGUGGCA  UCGAC CG CAGCACACCGU  miRNA 3' AC U U 5' |
|  | NM_001200064.1 | Ictalurus punctatus NLR family member X1 (nlrx1), mRNA | -31.7 | position 3097  target 5' A G U G A G 3'  GAGU GAGCG GU GUG GGCA  CUCG CUCGU CA CAC CCGU  miRNA 3' A A G A 5' |
|  | NM_001200547.1 | Ictalurus punctatus pdz and lim domain protein 3 (pdli3), mRNA | -25.2 | position 427  target 5' C C CCCUU G 3'  GGC GAGC G CG UGUGGCG  UCG CUCG C GC ACACCGU  miRNA 3' AC A U A 5' |
|  | NM_001257112.1 | Ictalurus punctatus protein arginine methyltransferase (prmt4), mRNA | -34.0 | position 205  target 5' A G A 3'  GAGU GCAGUCGUGUGG G  CUCG CGUCAGCACACC U  miRNA 3' A ACU G 5' |
|  | NM_001200263.1 | Ictalurus punctatus vertebrate ancient long opsin (LOC100305024), mRNA | -28.5 | position 93  target 5' C AAAA U C 3'  UGAG UGGGC UCGU UGGCA  ACUC ACUCG AGCA ACCGU  miRNA 3' G UC C 5' |
|  | NM_001200402.1 | Ictalurus punctatus serine/threonine-protein phosphatase 2a catalytic subunit beta isoform (pp2ab), mRNA | -25.0 | position 193  target 5' G C CCCUGUCA A 3'  UG GCUG CAGUU UGUGGCG  AC CGAC GUCAG ACACCGU  miRNA 3' U UC C 5' |
|  | NM_001201306.1 | Ictalurus punctatus isochorismatase domain containing 2 (isoc2), nuclear gene encoding mitochondrial protein, mRNA | -31.7 | position 346  target 5' G CCCA GCCAUAC U 3'  GAGC AGCAG UGUGUGGCA  CUCG UCGUC GCACACCGU  miRNA 3' A AC A 5' |
|  | NM_001201105.1 | Ictalurus punctatus canopy1 (cnpy1), mRNA | -33.7 | position 541  target 5' C GCGU CCAGA A 3'  GAGC GGC AGUUGUGUGGCG  CUCG UCG UCAGCACACCGU  miRNA 3' A AC 5' |
|  | NM_001200818.1 | Ictalurus punctatus cell differentiation protein rcd1-like protein (rcd1), mRNA | -32.3 | position 288  target 5' A CU C C 3'  UG CUG GCA UCGUGUGGCA  AC GAC CGU AGCACACCGU  miRNA 3' UC U C 5' |
| ipu-miR-7560 | NM_001200177.1 | Ictalurus punctatus inhibitor of apoptosis protein-1 (ciap-1), mRNA | -27.9 | position 1709  target 5' G CA C 3'  UCAAG GCAGACGGGU  AGUUC CGUCUGUCCA  miRNA 3' CGA UAA U 5' |
|  | NM_001200301.1 | Ictalurus punctatus hypoxia induced factor 1 alpha (LOC100305068), mRNA | -25.8 | position 2155  target 5' A G C UU A 3'  G UUCGAG UUGC GGCGGG  C AAGUUC AACG CUGUCC  miRNA 3' G U U AU 5' |
|  | NM_001200935.1 | Ictalurus punctatus metaxin 2 (mtx2), nuclear gene encoding mitochondrial protein, mRNA | -21.9 | position 582  target 5' G GGG AAGAGUC A U 3'  GCU CAGGA UUG ACAGGUG  CGA GUUCU AAC UGUCCAU  miRNA 3' A GUC 5' |
|  | NM_001200192.1 | Ictalurus punctatus thyroid stimulating hormone receptor (LOC100304626), mRNA | -25.3 | position 1689  target 5' A UGCCAU CUG A 3'  UCA AGA GCAGACAGG  AGU UCU CGUCUGUCC  miRNA 3' CGA AA AU 5' |
|  | NM_001200205.1 | Ictalurus punctatus liver-expressed antimicrobial peptide 2 (LOC100304644), mRNA | -24.1 | position 4  target 5' G G CUG U U 3'  U AAGA GCAGAC GGGUG  A UUCU CGUCUG UCCAU  miRNA 3' CGA G AA 5' |
|  | NM_001200184.1 | Ictalurus punctatus putative odorant receptor CF64 (LOC100304610), mRNA | -23.2 | position 1631  target 5' U CUGCAUCCCCUG G 3'  GCUUUGA UGCAGAUGGG  CGAAGUU ACGUCUGUCC  miRNA 3' CUA AU 5' |
|  | NM_001200189.1 | Ictalurus punctatus E-box binding protein 2 (LOC100304623), mRNA | -27.7 | position 2381  target 5' U AUC G A 3'  GCU CAAG GCAGACAGG  CGA GUUC CGUCUGUCC  miRNA 3' A UAA AU 5' |
|  | NM_001200318.1 | Ictalurus punctatus E-box binding protein 1 (eb1), mRNA | -27.7 | position 2370  target 5' U AUC G A 3'  GCU CAAG GCAGACAGG  CGA GUUC CGUCUGUCC  miRNA 3' A UAA AU 5' |
|  | NM_001200267.1 | Ictalurus punctatus olfactory receptor 1 (LOC100305028), mRNA | -22.8 | position 909  target 5' G CA U A 3'  GAGGU UGCAGGCGG UA  UUCUA ACGUCUGUC AU  miRNA 3' CGAAG C 5' |
|  | NM_001201088.1 | Ictalurus punctatus nicolin 1 (nicn1), mRNA | -25.4 | position 667  target 5' G CU C U U 3'  GCUUC GAU CAGACAG GUG  CGAAG CUA GUCUGUC CAU  miRNA 3' UU AC 5' |
| ipu-miR-7558b | NM_001200258.1 | Ictalurus punctatus annexin A4 (anxa4), mRNA | -22.4 | position 1288  target 5' G A 3'  GUGCUU UAAUCUCA  CACGAG GUUAGAGU  miRNA 3' CUCA G CGA 5' |
|  | NM_001200171.1 | Ictalurus punctatus Oct2 transcription factor (oct2), mRNA | -29.2 | position 768  target 5' U AG C U 3'  UG GC CUCAAUCUCAGCU  AC CG GGGUUAGAGUCGA  miRNA 3' CUC A A 5' |
|  | NM_001200222.1 | Ictalurus punctatus 6-pyruvoyl-tetrahydropterin synthase (LOC100304743), mRNA | -30.0 | position 77  target 5' A CAGC G C A 3'  GAG UGCUCCCAAU CUC GC  CUC ACGAGGGUUA GAG CG  miRNA 3' AC U A 5' |
|  | NM_001200711.1 | Ictalurus punctatus guanosine monophosphate reductase 2 (gmpr2), mRNA | -26.2 | position 549  target 5' A G AUCGG GG G 3'  AGUG GC UCCA CUCAGU  UCAC CG GGGU GAGUCG  miRNA 3' C A A UA A 5' |
|  | NM_001200546.1 | Ictalurus punctatus peroxisomal 3-ketoacyl-CoA thiolase A (thika), mRNA | -27.3 | position 635  target 5' U G C A 3'  UGCUU CAA UCUCAGC  ACGAG GUU AGAGUCG  miRNA 3' CUCAC G A 5' |
|  | NM_001200313.1 | Ictalurus punctatus steroidogenic cytochrome P450 17-hydroxylase/lyase (cyp17), mRNA | -30.2 | position 1132  target 5' A G C 3'  GUGCUCC AAUC CGGC  CACGAGG UUAG GUCG  miRNA 3' CUCA G A A 5' |
|  | NM_001200256.1 | Ictalurus punctatus insulin-like growth factor binding protein 3 (LOC100305016), mRNA | -26.3 | position 128  target 5' C A G GCG C 3'  GUGUG U CCGAU CUCGGC  CACAC A GGUUA GAGUCG  miRNA 3' CU G G A 5' |
| ipu-miR-7561 | NM_001200074.1 | Ictalurus punctatus estrogen receptor type alpha (er), mRNA | -28.6 | position 1520  target 5' C CAUAA U 3'  AGCGAGCUC AC UGAGUC  UCGCUCGAG UG ACUUAG  miRNA 3' U C AG U 5' |
|  | NM_001200228.1 | Ictalurus punctatus matrix metalloproteinase 9 (mmp9), mRNA | -27.6 | position 1743  target 5' C U ACUGCU G 3'  AGC GAGCUCGAC UUUGAG  UCG CUCGAGCUG AGACUU  miRNA 3' U AGU 5' |
|  | NM_001200301.1 | Ictalurus punctatus hypoxia induced factor 1 alpha (LOC100305068), mRNA | -27.6 | position 2326  target 5' G U U C A 3'  GAGC AGC UCG GCUCUGGA CA  UUCG UCG AGC UGAGACUU GU  miRNA 3' C A 5' |
|  | NM_001200313.1 | Ictalurus punctatus steroidogenic cytochrome P450 17-hydroxylase/lyase (cyp17), mRNA | -26.3 | position 1566  target 5' A CAA G G 3'  GAGU GA UCUGAGUCA  CUCG CU AGACUUAGU  miRNA 3' UUCG AG G 5' |
|  | NM_001200361.1 | Ictalurus punctatus adaptor-related protein complex 3, sigma 2 subunit (ap3s2), mRNA | -37.2 | position 304  target 5' A AU CC U 3'  GAGCGAGCUCGGC UCUGGA UCA  UUCGCUCGAGCUG AGACUU AGU  miRNA 3' 5' |
|  | NM_001200785.1 | Ictalurus punctatus vacuolar protein sorting 28 (yeast) (vps28), mRNA | -23.2 | position 115  target 5' G CAGCCAAUAA U 3'  GAG GAGCUC GC CUGAAUUA  UUC CUCGAG UG GACUUAGU  miRNA 3' G C A 5' |
| ipu-miR-7562 | NM_001201090.1 | Ictalurus punctatus 2-oxoglutarate/malate carrier protein, nuclear gene encoding mitochondrial protein, mRNA | -34.9 | position 1077  target 5' G U UC U 3'  UGUGUGUUCGUG GU GUGUGUG  ACACACAAGUAC CA CACACAC  miRNA 3' U 5' |
|  | NM_001200876.1 | Ictalurus punctatus dihydrofolate reductase (dyr), mRNA | -32.1 | position 175  target 5' A A G G 3'  GUG GU UGUGAGUGUGUGUG  CAC CA GUACUCACACACAC  miRNA 3' A A A 5' |
|  | NM_001200677.1 | Ictalurus punctatus upf0428 protein cxorf56-like protein (cx056), mRNA | -33.3 | position 771  target 5' G GUG U U 3'  UGUGUGU UGUG GUGUGUGUG  ACACACA GUAC CACACACAC  miRNA 3' A U 5' |
|  | NM_001200455.1 | Ictalurus punctatus glyoxylate reductase/hydroxypyruvate reductase (grhpr), mRNA | -32.0 | position 45  target 5' G G UUC A 3'  UGUGUGU UCGUG GUGUGUGU  ACACACA AGUAC CACACACA  miRNA 3' U C 5' |
|  | NM_001200432.1 | Ictalurus punctatus tRNA (guanine-n(7)-)-methyltransferase b (trmbb), mRNA | -29.3 | position 104  target 5' C GGAG GGA A 3'  UG GUUCA UGAGUGUGUGU  AC CAAGU ACUCACACACA  miRNA 3' AC A C 5' |
|  | NM_001200312.1 | Ictalurus punctatus cytochrome P450 cholesterol side chain cleavage (cyp11a), mRNA | -33.4 | position 124  target 5' A G U G 3'  GUGUGU UGUG GUGUGUGUG  CACACA GUAC CACACACAC  miRNA 3' A A U 5' |
|  | NM_001201023.1 | Ictalurus punctatus phosphoribosyltransferase domain-containing protein 1 (prdc1), mRNA | -33.3 | position 810  target 5' G GUG U U 3'  UGUGUGU UGUG GUGUGUGUG  ACACACA GUAC CACACACAC  miRNA 3' A U 5' |
|  | NM_001200749.1 | Ictalurus punctatus uncharacterized protein c1orf109-like protein (ca109), mRNA | -26.5 | position 177  target 5' C UA UAGCC G 3'  GU GUUU GAGUGUGUGUG  CA CAAG CUCACACACAC  miRNA 3' A CA UA 5' |
|  | NM_001200633.1 | Ictalurus punctatus DNA replication complex gins protein psf3 (psf3), mRNA | -33.8 | position 760  target 5' G GUG U A 3'  UGUGUGU UGUG GUGUGUGUG  ACACACA GUAC CACACACAC  miRNA 3' A U 5' |
|  | NM_001257113.1 | Ictalurus punctatus protein arginine methyltransferase (co-prmt), mRNA | -25.4 | position 1495  target 5' C G AC U 3'  UGUGUU CAU GUGUGUGU  ACACAA GUA CACACACA  miRNA 3' AC CU C 5' |
|  | NM_001257111.1 | Ictalurus punctatus protein arginine methyltransferase 1 (prmt1), mRNA | -33.7 | position 1107  target 5' C G U G 3'  UGUGUGU UGUG GUGUGUGUG  ACACACA GUAC CACACACAC  miRNA 3' A U 5' |
|  | NM_001201118.1 | Ictalurus punctatus charged multivesicular body protein 1b (chm1b), mRNA | -36.2 | position 769  target 5' G GUG U A 3'  UGUGUGU CGUG GUGUGUGUG  ACACACA GUAC CACACACAC  miRNA 3' A U 5' |
|  | NM_001201012.1 | Ictalurus punctatus transmembrane protein 179b (t179b), mRNA | -23.1 | position 193  target 5' C CAGG CUCU A 3'  GUGU CAU GUGUGUGU  CACA GUA CACACACA  miRNA 3' A CAA CU C 5' |
|  | NM_001200962.1 | Ictalurus punctatus ribonuclease h2 subunit a (rnh2a), mRNA | -30.9 | position 902  target 5' G GUCUG UGUAU U 3'  UGUGUGU UAUG GUGUGUGUG  ACACACA GUAC CACACACAC  miRNA 3' A U 5' |
|  | NM_001200928.1 | Ictalurus punctatus inhibitor of DNA binding 1 (id1), mRNA | -33.8 | position 521  target 5' G GUG U A 3'  UGUGUGU UGUG GUGUGUGUG  ACACACA GUAC CACACACAC  miRNA 3' A U 5' |
|  | NM_001200780.1 | Ictalurus punctatus monocyte to macrophage differentiation protein (paqrb), mRNA | -33.8 | position 1001  target 5' G GUG U A 3'  UGUGUGU UGUG GUGUGUGUG  ACACACA GUAC CACACACAC  miRNA 3' A U 5' |
|  | NM_001200755.1 | Ictalurus punctatus ras-related protein m-ras (rasm), mRNA | -35.7 | position 184  target 5' G GUG U U 3'  UGUGUGU CGUG GUGUGUGUG  ACACACA GUAC CACACACAC  miRNA 3' A U 5' |
|  | NM_001200260.1 | Ictalurus punctatus annexin A11 (LOC100305021), mRNA | -33.3 | position 1615  target 5' G GUG U U 3'  UGUGUGU UGUG GUGUGUGUG  ACACACA GUAC CACACACAC  miRNA 3' A U 5' |
|  | NM_001200558.1 | Ictalurus punctatus v-set and transmembrane domain-containing protein 2b (vtm2b), mRNA | -33.3 | position 127  target 5' G GUG U U 3'  UGUGUGU UGUG GUGUGUGUG  ACACACA GUAC CACACACAC  miRNA 3' A U 5' |
|  | NM_001200350.1 | Ictalurus punctatus breast cancer metastasis-suppressor 1-like protein (brm1l), mRNA | -33.8 | position 1217  target 5' G GUG U A 3'  UGUGUGU UGUG GUGUGUGUG  ACACACA GUAC CACACACAC  miRNA 3' A U 5' |
|  | NM_001200090.1 | Ictalurus punctatus oncoprotein myc (myc), mRNA | -35.1 | position 19  target 5' C G U U 3'  GUGUGU CGUG GUGUGUGUG  CACACA GUAC CACACACAC  miRNA 3' A A U 5' |
|  | NM_001200787.1 | Ictalurus punctatus c-myc binding protein (mycbp), mRNA | -33.6 | position 506  target 5' G G U A 3'  UGUGUGU UGUG GUGUGUGUG  ACACACA GUAC CACACACAC  miRNA 3' A U 5' |
| ipu-miR-7563a | NM_001200281.1 | Ictalurus punctatus ribosomal protein L5b (LOC100305046), mRNA | -25.4 | position 432  target 5' A GAC GAGUUUA A 3'  GGU GGGCGAG ACGUGG  UCA UCCGUUC UGCGCC  miRNA 3' AUA A AA 5' |
|  | NM_001201128.1 | Ictalurus punctatus solute carrier family 25 member 44 (s2544), mRNA | -23.1 | position 517  target 5' C UUUACAG A C 3'  GUGGGU AGG UACGUGG  CAUCCG UUC AUGCGCC  miRNA 3' AUAU AA 5' |
|  | NM_001201081.1 | Ictalurus punctatus caspase-3 (casp3), mRNA | -28.3 | position 66  target 5' C C G 3'  GUGGG AAGUACG CGGU  CAUCC UUCAUGC GCCA  miRNA 3' AUAU G A 5' |
| ipu-miR-7563b | NM_001201085.1 | Ictalurus punctatus mitochondrial putative octanoyltransferase (lipb), mRNA | -24.6 | position 554  target 5' U A UCC U A 3'  GUGCA UAGGUA AC GUGGU  UAUGU AUCCAU UG CGCCA  miRNA 3'G C UCA 5' |
|  | NM_001200472.1 | Ictalurus punctatus tubulin beta-2 chain (tbb2), mRNA | -27.2 | position 193  target 5' C GC A C 3'  UACGG GG AAGUACGUG  AUGUC CC UUCAUGCGC  miRNA 3'GU AU A CA 5' |
|  | NM_001200311.1 | Ictalurus punctatus complement factor I (cfi), mRNA | -24.3 | position 1350  target 5' G GGC C A A 3'  AUACAGU AGGUGG UGUGC GG  UAUGUCA UCCAUU AUGCG CC  miRNA 3'G C A 5' |
|  | NM_001200083.1 | Ictalurus punctatus estrogen receptor type beta (LOC100304489), mRNA | -28.1 | position 1351  target 5' C A AG U C 3'  UACAG AGG GAGUACGUG GU  AUGUC UCC UUCAUGCGC CA  miRNA 3'GU A A 5' |
| ipu-miR-7564 | NM_001200066.1 | Ictalurus punctatus NLR family, CARD domain containing 5 (nlrc5), mRNA | -36.8 | position 702  target 5' U A AGG U 3'  GCUGUC GGUC CUGGCUCUGG  CGACAG CUAG GACCGAGACU  miRNA 3' A UU 5' |
|  | NM_001201201.1 | Ictalurus punctatus mitochondrial malate dehydrogenase, nuclear gene encoding mitochondrial protein, mRNA | -26.5 | position 313  target 5' A GG C G 3'  GCUG CG GGCUCUGAAA  CGAC GC CCGAGACUUU  miRNA 3' A UAGAGA 5' |
|  | NM_001201171.1 | Ictalurus punctatus nf-kappa-b inhibitor-interacting ras-like protein 2 (kbrs2), mRNA | -26.1 | position 502  target 5' G C AG GAAAG A G 3'  GCU GUC GA UC GGCUCUGGGA  CGA CAG CU AG CCGAGACUUU  miRNA 3' AG A 5' |
|  | NM_001245943.1 | Ictalurus punctatus creatine kinase (LOC100304493), mRNA | -28.5 | position 528  target 5' A G GAAGC G UUGAA C 3'  GCUGU GA U UCUG GCUCUGAA  CGACA CU A AGAC CGAGACUU  miRNA 3' G G U 5' |
|  | NM_001201295.1 | Ictalurus punctatus eukaryotic translation initiation factor 3, subunit M (eif3m), mRNA | -32.4 | position 701  target 5' U U CCA C C 3'  CUG UUGA UCU CUGGCUCUGAAA  GAC AGCU AGA GACCGAGACUUU  miRNA 3' C 5' |
|  | NM_001200100.1 | Ictalurus punctatus natural resistance-associated macrophage protein large transcript (LOC100304514), mRNA | -29.9 | position 547  target 5' A G C A C 3'  GCUG C AUC UUGGCUCUGA  CGAC G UAG GACCGAGACU  miRNA 3' A C A UU 5' |
| ipu-miR-7563c | NM_001201085.1 | Ictalurus punctatus mitochondrial putative octanoyltransferase (lipb), mRNA | -23.9 | position 554  target 5' U A UCC U A 3'  GUGCA UAGGUA AC GUGGU  UAUGU AUCCAU UG CGCCA  miRNA 3' C UCA 5' |
|  | NM_001200472.1 | Ictalurus punctatus tubulin beta-2 chain (tbb2), mRNA | -27.2 | position 193  target 5' C GC A C 3'  UACGG GG AAGUACGUG  AUGUC CC UUCAUGCGC  miRNA 3' U AU A CA 5' |
|  | NM_001200311.1 | Ictalurus punctatus complement factor I (cfi), mRNA | -23.6 | position 1350  target 5' G GGC C A A 3'  AUACAGU AGGUGG UGUGC GG  UAUGUCA UCCAUU AUGCG CC  miRNA 3' C A 5' |
|  | NM_001200083.1 | Ictalurus punctatus estrogen receptor type beta (LOC100304489), mRNA | -28.1 | position 1351  target 5' C A AG U C 3'  UACAG AGG GAGUACGUG GU  AUGUC UCC UUCAUGCGC CA  miRNA 3' U A A 5' |
| ipu-miR-7565 | NM_001201265.1 | Ictalurus punctatus proteasome (prosome, macropain) 26S subunit, non-ATPase, 6 (psmd6), mRNA | -28.4 | position 915  target 5' U C C GG A 3'  CU GCUCA GU AGCAGGAA  GA CGAGU CA UCGUCCUU  miRNA 3' U C AG 5' |
|  | NM_001200958.1 | Ictalurus punctatus uncharacterized protein c22orf25 (cv025), mRNA | -28.1 | position 396  target 5' G GG U A 3'  ACUGGU GG C GCGGGAA  UGACCG UC G CGUCCUU  miRNA 3' AG AA U 5' |
|  | NM_001200411.1 | Ictalurus punctatus deoxyhypusine synthase (dhys), mRNA | -28.5 | position 263  target 5' G AGC GGAGG A 3'  ACUGG CGGU AGCAGGAA  UGACC GUCA UCGUCCUU  miRNA 3' GA AG 5' |
|  | NM_001200301.1 | Ictalurus punctatus hypoxia induced factor 1 alpha (LOC100305068), mRNA | -28.7 | position 1273  target 5' U CAG U A 3'  CUGGCUC C GCAGGGG  GACCGAG G CGUCCUU  miRNA 3' U UCAA U 5' |
|  | NM_001200184.1 | Ictalurus punctatus putative odorant receptor CF64 (LOC100304610), mRNA | -25.9 | position 1580  target 5' A U CCA GAC C 3'  ACUG CUC GG CAGCAGGG  UGAC GAG UC GUCGUCCU  miRNA 3' C AA U 5' |
|  | NM_001200553.1 | Ictalurus punctatus methylmalonic aciduria and homocystinuria type c-like protein (mmac), mRNA | -33.9 | position 364  target 5' U CUGCGGCUCAUG C 3'  CUGGCUCAGU UAGCAGGAG  GACCGAGUCA GUCGUCCUU  miRNA 3' U A 5' |
|  | NM_001200091.1 | Ictalurus punctatus follicle-stimulating hormone receptor (LOC100304505), mRNA | -29.1 | position 179  target 5' C UGAUG C A 3'  UUGGCUCA CA GCAGGGA  GACCGAGU GU CGUCCUU  miRNA 3' U CAA 5' |
| ipu-miR-7566 | GU589292.1 | Ictalurus punctatus clone CBZB29983 pq-loop repeat-containing protein 2 (PQLC2) mRNA, complete cds | -25.8 | position 759  target 5' G CUUUCUGUUC C U 3'  GCGUGUC GGU G GCUGGUGA  CGCACAG CUA C CGGCUACU  miRNA 3' A U 5' |
|  | NM_001200212.1 | Ictalurus punctatus hypoxia induced factor-like factor (LOC100304653), mRNA | -27.2 | position 1438  target 5' C A GCUC G 3'  UG UCUGA GGCCGAUGA  AC AGACU UCGGCUACU  miRNA 3' CGC AC 5' |
|  | NM_001200585.1 | Ictalurus punctatus RAS related protein 1b (rap1b), mRNA | -29.6 | position 344  target 5' U A AAAGCCACCGA U U 3'  GCG GUU UGAUG GCCGAUGA  CGC CAG ACUAC CGGCUACU  miRNA 3' A U 5' |
|  | NM_001200189.1 | Ictalurus punctatus E-box binding protein 2 (LOC100304623), mRNA | -24.8 | position 299  target 5' C A UCGCC A 3'  GCG UGUUU GCCGGUGA  CGC ACAGA CGGCUACU  miRNA 3' CUACU 5' |
|  | NM_001200295.1 | Ictalurus punctatus insulin-like growth factor I (igf-i), mRNA | -23.6 | position 523  target 5' C CUCGCG C C 3'  GCG CUGA GCCGGUGG  CGC GACU CGGCUACU  miRNA 3' ACA ACU 5' |
| ipu-miR-7567 | NM_001200066.1 | Ictalurus punctatus NLR family, CARD domain containing 5 (nlrc5), mRNA | -28.2 | position 1620  target 5' G AGAUG CGGC U 3'  UGG GAUCG GUCUGGGC  ACC CUAGC UAGACCCG  miRNA 3' UCG G UU 5' |
|  | NM_001201154.1 | Ictalurus punctatus carboxy-terminal domain RNA polymerase II polypeptide a small phohatase 1, mRNA | -32.0 | position 835  target 5' C A C G C 3'  AGCU G GUGAUCGA C GGGCAG  UCGA C CGCUAGCU G CCCGUU  miRNA 3' A A 5' |
|  | NM_001200100.1 | Ictalurus punctatus natural resistance-associated macrophage protein large transcript (LOC100304514), mRNA | -34.6 | position 1660  target 5' U A UGUCUGA C U 3'  GGC UGGCGG UCG UCUGGGCG  UCG ACCGCU AGC AGACCCGU  miRNA 3' U U 5' |
|  | NM_001200312.1 | Ictalurus punctatus cytochrome P450 cholesterol side chain cleavage (cyp11a), mRNA | -28.2 | position 1543  target 5' G UUG UU G C 3'  AGU GGC U GAUUUGGGC  UCG CCG A CUAGACCCG  miRNA 3' A CU G UU 5' |
|  | NM_001200878.1 | Ictalurus punctatus spliceosome RNA helicase bat1 (uap56), mRNA | -26.5 | position 1202  target 5' U C U C AAC G 3'  GCU G G CGGUCGAUUUGG CAA  CGA C C GCUAGCUAGACC GUU  miRNA 3' U C 5' |
| ipu-miR-7568 | NM_001200310.1 | Ictalurus punctatus mammal-like melanopsin 2 (opn4m2), mRNA | -26.2 | position 476  target 5' C C GUUAUCG U 3'  UCA UAC GGUCGGUCA  AGU GUG CCAGCCAGU  miRNA 3' UA C AA C 5' |
|  | NM_001200177.1 | Ictalurus punctatus inhibitor of apoptosis protein-1 (ciap-1), mRNA | -27.8 | position 853  target 5' C UG C 3'  UUCAGC U GGU GGUCA  AAGUCG A CCA CCAGU  miRNA 3' U UG A G C 5' |
|  | NM_001200090.1 | Ictalurus punctatus oncoprotein myc (myc), mRNA | -23.7 | position 510  target 5' C ACGACG A 3'  UCGGCG CGGUCA  AGUCGU GCCAGU  miRNA 3' UA GAACCA C 5' |
|  | NM_001200663.1 | Ictalurus punctatus ADP-ribosylarginine hydrolase (adprh), mRNA | -26.7 | position 945  target 5' A U 3'  CAGCAC GG CGGUCA  GUCGUG CC GCCAGU  miRNA 3' UAA AA A C 5' |
| ipu-miR-7569 | NM_001257115.1 | Ictalurus punctatus complement factor B/C2B (LOC100862743), mRNA | -22.2 | position 2314  target 5' C UC GAAAUACA U 3'  UGGAGGA ACA AGGAUUAUA  ACCUCUU UGU UUCUAAUAU  miRNA 3' AG 5' |
|  | NM_001200366.1 | Ictalurus punctatus nipsnap-like 2 (nips2), mRNA | -20.2 | position 830  target 5' A UC A 3'  UGGAG CAGGGUUAUG  ACCUC GUUCUAAUAU  miRNA 3' UUUGUA 5' |
| ipu-miR-7570 | NM_001201090.1 | Ictalurus punctatus 2-oxoglutarate/malate carrier protein, nuclear gene encoding mitochondrial protein, mRNA | -27.1 | position 842  target 5' C C AAGGUG A 3'  GA GUGCUGGUG GUGAGGA  CU CACGGCCGC UAUUCUU  miRNA 3' U GUG 5' |
|  | NM_001200164.1 | Ictalurus punctatus tyrosylprotein sulfotransferase-2 (LOC100304582), mRNA | -29.0 | position 927  target 5' G AGUUCCUGAAA ACCAUG G 3'  AGAGUGCUGG GU GCAUGAGGG  UCUCACGGCC CG UGUAUUCUU  miRNA 3' G 5' |
|  | NM_001200076.1 | Ictalurus punctatus tumor protein p53 (tp53), mRNA | -28.3 | position 960  target 5' U A UU G 3'  GG GC GGCGCACGU GAA  UC CG CCGCGUGUA CUU  miRNA 3' UC A G UU 5' |
| ipu-miR-7571 | NM_001200067.1 | Ictalurus punctatus nucleotide-binding oligomerization domain containing 1 (nod1), mRNA | -29.5 | position 1473  target 5' G ACU U C G 3'  AGGG UGGUGUC GUG AGCU  UCCC ACCACAG UAC UCGG  miRNA 3' AG A AC 5' |
|  | NM_001200965.1 | Ictalurus punctatus mitochondrial creatine kinase s-type (kcrs), nuclear gene encoding mitochondrial protein, mRNA | -32.5 | position 818  target 5' G A C AU A 3'  CAGGG UGG UCGUG UGGCCUG  GUCCC ACC AGUAC AUCGGAC  miRNA 3' A AC 5' |
|  | NM_001200320.1 | Ictalurus punctatus transferrin (LOC100335020), mRNA | -27.9 | position 1572  target 5' U GUGUUUGGCUGAA CG G C U 3'  UCAG GGUGG GU A GUGGCCU  AGUC CCACC CA U CAUCGGA  miRNA 3' A G A C 5' |
| ipu-miR-7572 | NM_001201210.1 | Ictalurus punctatus eukaryotic translation initiation factor 6 (if6), mRNA | -25.2 | position 1045  target 5' C UUUAUUG A 3'  AACAUU GUAAAGCUGCAG  UUGUGA CAUUUCGACGUU  miRNA 3' CCU AG 5' |
|  | NM_001201110.1 | Ictalurus punctatus DNA-directed RNA polymerase III subunit rpc6 (rpc6), mRNA | -27.1 | position 1  target 5' CUAU G 3'  GGGGACGCUG GUUGUAGU  CCUUUGUGAC CGACGUUA  miRNA 3' AUUU G 5' |
|  | NM_001200711.1 | Ictalurus punctatus guanosine monophosphate reductase 2 (gmpr2), mRNA | -24.5 | position 975  target 5' U UGCUAA AA G 3'  GGGAGC GCUG GGAGCUG AGUC  CCUUUG UGAC UUUCGAC UUAG  miRNA 3' A G |
|  | NM_001200385.1 | Ictalurus punctatus DMC1 dosage suppressor of mck1 homolog, mRNA | -27.2 | position 324  target 5' A U G U 3'  GGAAGC GCUG GAAGUUGC  CCUUUG UGAC UUUCGACG  miRNA 3' A UUAG 5' |
|  | NM_001200098.1 | Ictalurus punctatus novel immune-type receptor 7 (nitr7), mRNA | -21.3 | position 375  target 5' U GU U 3'  GGAACAC CUGCAAUU  CUUUGUG GACGUUAG  miRNA 3' C ACAUUUC 5' |
| ipu-miR-7573 | NM_001201105.1 | Ictalurus punctatus canopy1 (cnpy1), mRNA | -31.3 | position 138  target 5' A CUACU G 3'  AGUGCU GCUCAGCCU  UCACGG CGAGUCGGA  miRNA 3' GAG UAGUC 5' |
|  | NM_001200206.1 | Ictalurus punctatus E2A2 transcription factor (LOC100304646), mRNA | -32.0 | position 728  target 5' A CC G A 3'  UCAG GCU U GGGCUCAGC  AGUC CGG A UCCGAGUCG  miRNA 3' G A U G GA 5' |
|  | NM_001200352.1 | Ictalurus punctatus leucine zipper and CTNNBIP1 domain containing (lzic), mRNA | -31.6 | position 718  target 5' G C U A G 3'  UCAGUGCCA UC G GC CAGUUU  AGUCACGGU AG C CG GUCGGA  miRNA 3' G U A 5' |
|  | NM_001200170.1 | Ictalurus punctatus Oct1 transcription factor (oct1), mRNA | -31.3 | position 150  target 5' G UGUUUCU AG A 3'  CAGU GC CAGGCUCAGC  GUCA CG GUCCGAGUCG  miRNA 3' GA GUA GA 5' |
|  | NM_001201106.1 | Ictalurus punctatus TOB1 protein (tob1), mRNA | -28.0 | position 990  target 5' G CUCG G U C 3'  CUCA GCC U AG CUCAGCC  GAGU CGG A UC GAGUCGG  miRNA 3' CA U G C A 5' |
|  | NM_001200085.1 | Ictalurus punctatus tyrosinase (LOC100304491), mRNA | -41.6 | position 1148  target 5' C A A 3'  CUCAGUGC GGGCUCAGCC  GAGUCACG UCCGAGUCGG  miRNA 3' GUAG A 5' |
|  | NM_001200081.1 | Ictalurus punctatus CD18 (LOC100304487), mRNA | -29.1 | position 650  target 5' G CCAGCAUUUG UCAUGUC G 3'  UCAG GCUAUCA CUCAGCCU  AGUC CGGUAGU GAGUCGGA  miRNA 3' G A CC 5' |
|  | NM_001200906.1 | Ictalurus punctatus adenylate kinase 2 (kad2), nuclear gene encoding mitochondrial protein, mRNA | -28.9 | position 683  target 5' U CCA GAUGCU A 3'  UCA UGCCAUU GCUCAGUC  AGU ACGGUAG CGAGUCGG  miRNA 3' G C UC A 5' |
|  | NM_001200279.1 | Ictalurus punctatus luteinizing hormone receptor (LOC100305044), mRNA | -29.8 | position 47  target 5' U U G 3'  GGUGUCA CGG GCUCGGU  UCACGGU GUC CGAGUCG  miRNA 3' GAG A GA 5' |
|  | NM_001200083.1 | Ictalurus punctatus estrogen receptor type beta (LOC100304489), mRNA | -31.7 | position 1145  target 5' G C A G 3'  CUUGG CCAUCAGGU CAGCU  GAGUC GGUAGUCCG GUCGG  miRNA 3' AC A A 5' |
| ipu-miR-7574 | NM_001201177.1 | Ictalurus punctatus transmembrane protein 185a (t185a), mRNA | -21.9 | position 545  target 5' U C U ACUG U U 3'  GAC G GC GAG UGGAGAUAA  CUG C CG CUC AUCUCUAUU  miRNA 3' U U U 5' |
|  | NM_001200066.1 | Ictalurus punctatus NLR family, CARD domain containing 5 (nlrc5), mRNA | -24.5 | position 141  target 5' U UG CAUUUUGU CAG U 3'  AG GA GUGAG AGAGAUAA  UC CU CGCUC UCUCUAUU  miRNA 3' UG A U 5' |
|  | NM_001200844.1 | Ictalurus punctatus proteasome (prosome, macropain) assembly chaperone 2 (psmg2), mRNA | -24.7 | position 545  target 5' G CU A GCU G 3'  GGACG GC GAG GGGAUGGA  UCUGC CG CUC CUCUAUUU  miRNA 3' U AU 5' |
|  | NM_001200560.1 | Ictalurus punctatus N-myc downstream regulated gene 2 (ndrg2), mRNA | -29.2 | position 1330  target 5' G GA AG U 3'  AGA GAGCGAG AGAGGUGGA  UCU CUCGCUC UCUCUAUUU  miRNA 3' G A 5' |
|  | GU589292.1 | Ictalurus punctatus clone CBZB29983 pq-loop repeat-containing protein 2 (PQLC2) mRNA, complete cds | -25.5 | position 43  target 5' U ACUCUCAGACAU CAGAU U 3'  GACGAGCGA GU GGGGAUGG  CUGCUCGCU CA UCUCUAUU  miRNA 3' U U 5' |
|  | NM_001200340.1 | Ictalurus punctatus 26S proteasome non-ATPase regulatory subunit 10 (psd10), mRNA | -31.8 | position 579  target 5' U G C 3'  GA GAGCGAGUAGAGG  CU CUCGCUCAUCUCU  miRNA 3' U G AUUU 5' |
|  | NM_001200988.1 | Ictalurus punctatus stomatin (stom), mRNA | -35.7 | position 659  target 5' A UG U 3'  GG GAGCGAGUGGAGAU  UC CUCGCUCAUCUCUA  miRNA 3' UG UUU 5' |
|  | NM_001200715.1 | Ictalurus punctatus transmembrane protein 45b (tm45b), mRNA | -23.5 | position 1070  target 5' A AAC U 3'  AUGAG AGUAGAGAUG  UGCUC UCAUCUCUAU  miRNA 3' UC GC UU 5' |
| ipu-miR-7575 | NM_001200672.1 | Ictalurus punctatus MIT, microtubule interacting and transport, domain containing 1 (mitd1), mRNA | -25.5 | position 1114  target 5' A C A 3'  ACUAUGA CAUG CUGUGC  UGGUACU GUAC GGUACG  miRNA 3' C A U 5' |
|  | NM_001201102.1 | Ictalurus punctatus lissencephaly-1-like protein A (lis1a), mRNA | -28.2 | position 566  target 5' G CAUGGG A 3'  GACCAUG CAUGACCAU  CUGGUAC GUACUGGUA  miRNA 3' UA CG 5' |
|  | NM_001201128.1 | Ictalurus punctatus solute carrier family 25 member 44 (s2544), mRNA | -27.7 | position 53  target 5' U C U 3'  GACCAUGA CAU CCGUGC  CUGGUACU GUA GGUACG  miRNA 3' A CU 5' |
|  | NM_001200548.1 | Ictalurus punctatus elongation of very long chain fatty acids protein 6 (elov6), mRNA | -26.6 | position 507  target 5' C GU A 3'  GGCUG UCAUGACCAUG  CUGGU AGUACUGGUAC  miRNA 3' ACU G 5' |
|  | NM_001201284.1 | Ictalurus punctatus uncharacterized protein c17orf72-like protein (cq072), mRNA | -28.7 | position 243  target 5' C ACAA C 3'  AUCAUGA CAUGACCAUGU  UGGUACU GUACUGGUACG  miRNA 3' C A 5' |
|  | NM_001200364.1 | Ictalurus punctatus transmembrane protein 198 (tm198), mRNA | -25.7 | position 545  target 5' U C A 3'  GCCAU AUCAUGAC AUGU  UGGUA UAGUACUG UACG  miRNA 3' C C G 5' |
|  | NM_001201312.1 | Ictalurus punctatus ADP-ribosylation factor-like 4a (arl4a), mRNA | -26.2 | position 823  target 5' G AUU G 3'  UCAUGAU UGACCAUG  GGUACUA ACUGGUAC  miRNA 3' CU GU G 5' |
|  | NM_001200073.1 | Ictalurus punctatus parapinopsin (LOC100304471), mRNA | -31.2 | position 741  target 5' C UUUU U 3'  AUCAUGGUCAUGGCC UGC  UGGUACUAGUACUGG ACG  miRNA 3' C U 5' |
|  | NM_001200764.1 | Ictalurus punctatus 14-3-3 protein-like protein 2 (14332), mRNA | -22.9 | position 105  target 5' C U GGA U A 3'  GCU UGA UAUGGCCGU GC  UGG ACU GUACUGGUA CG  miRNA 3' C U A 5' |
|  | NM_001200279.1 | Ictalurus punctatus luteinizing hormone receptor (LOC100305044), mRNA | -26.6 | position 1452  target 5' C CAC A 3'  ACCAU UCAUG CCAUGC  UGGUA AGUAC GGUACG  miRNA 3' C CU U 5' |
|  | NM_001200260.1 | Ictalurus punctatus annexin A11 (LOC100305021), mRNA | -27.0 | position 1344  target 5' G C CG G 3'  GACU UGAUC GAUCAUG  CUGG ACUAG CUGGUAC  miRNA 3' U UA G 5' |
|  | NM_001200853.1 | Ictalurus punctatus prostaglandin reductase 1 (ptgr1), mRNA | -25.8 | position 808  target 5' C U 3'  CCA CAUGACCAUGC  GGU GUACUGGUACG  miRNA 3' CU ACUA 5' |
|  | NM_001200604.1 | Ictalurus punctatus aminoacyl tRNA synthetase complex-interacting multifunctional protein 1, mRNA | -31.5 | position 691  target 5' A UGU A 3'  GGCCAUGGUCAUG GCCA GC  CUGGUACUAGUAC UGGU CG  miRNA 3' A 5' |
|  | NM_001200838.1 | Ictalurus punctatus transmembrane protein 106b (t106b), mRNA | -23.6 | position 69  target 5' A G 3'  GGCCA GGU GUGAUCAUG  CUGGU CUA UACUGGUAC  miRNA 3' A G G 5' |
| ipu-miR-7576 | NM_001200722.1 | Ictalurus punctatus pelota homolog (Drosophila) (pelo), mRNA | -31.1 | position 947  target 5' A GCU G 3'  UG GCGGCGGUUGAG UUC  AC CGCCGCCAACUU AAG  miRNA 3' A AC A 5' |
|  | NM_001200694.1 | Ictalurus punctatus rho-related gtp-binding protein rhoc (rhoc), mRNA | -24.9 | position 551  target 5' G GA G 3'  GUGU GCGG GGUGUUC  CACG CGCC UUACAAG  miRNA 3' A C AAC A 5' |
| ipu-miR-7577 | NM_001201265.1 | Ictalurus punctatus proteasome (prosome, macropain) 26S subunit, non-ATPase, 6 (psmd6), mRNA | -27.9 | position 574  target 5' A AAAG U 3'  CCGAG CCAAGA GUCUGA  GGCUC GGUUUU CAGGCU  miRNA 3' A A A C 5' |
|  | NM_001201131.1 | Ictalurus punctatus mll1/mll complex subunit c17orf49-like protein (cq049), mRNA | -25.5 | position 704  target 5' C A U 3'  UUCGA UC AGAGUGUCUGAG  AGGCU AG UUUUACAGGCUC  miRNA 3' C G 5' |
|  | NM_001201136.1 | Ictalurus punctatus mosc domain-containing protein 1, nuclear gene encoding mitochondrial protein, mRNA | -27.0 | position 691  target 5' C U AUGUU U 3'  UCC GUC GAUGUCCGAG  AGG CAG UUACAGGCUC  miRNA 3' CU GUU 5' |
|  | NM_001201083.1 | Ictalurus punctatus transmembrane protein 120b (t120b), mRNA | -23.8 | position 106  target 5' G CAU A C 3'  GAGUCU AAGGUGU CCGG  CUCAGG UUUUACA GGCU  miRNA 3' AGG C 5' |
|  | NM_001200207.1 | Ictalurus punctatus leukocyte immune-type receptor 3 (LOC100304647), mRNA | -31.4 | position 2149  target 5' A AG G A 3'  UCCGAGUC UAAAAU GUCCGA  AGGCUCAG GUUUUA CAGGCU  miRNA 3' C 5' |
|  | NM_001201315.1 | Ictalurus punctatus carboxypeptidase a1 (cbpa1), mRNA | -24.6 | position 190  target 5' G CUG UGUC U 3'  UCG CC GAUGUCCGAG  GGC GG UUACAGGCUC  miRNA 3' A UCA UU 5' |
| ipu-miR-457b | NM_001200264.1 | Ictalurus punctatus mammal-like melanopsin 1 (opn4m1), mRNA | -22.7 | position 378  target 5' G CUCUUCGG U 3'  UGCU GAUGUGCU CUA  ACGG CUACACGA GAU  miRNA 3' UUAUAA C 5' |
|  | NM_001200213.1 | Ictalurus punctatus factor inhibiting HIF-1 (LOC100304654), mRNA | -26.3 | position 6  target 5' G G GCGCA C 3'  GC AG GUGUGCUGCUG  CG UU UACACGACGAU  miRNA 3' A G AUAAC 5' |
|  | NM_001200184.1 | Ictalurus punctatus putative odorant receptor CF64 (LOC100304610), mRNA | -23.4 | position 1620  target 5' A U U 3'  GCCAAU UGCUGCU  CGGUUA ACGACGA  miRNA 3' A UAACUAC U 5' |
|  | NM_001200174.1 | Ictalurus punctatus Mx1 protein (mx1), mRNA | -25.1 | position 1838  target 5' C C AUCAGGUACC C 3'  UGCCG UGGUG UGCUGCUG  ACGGU ACUAC ACGACGAU  miRNA 3' UAUA 5' |
|  | NM_001200100.1 | Ictalurus punctatus natural resistance-associated macrophage protein large transcript (LOC100304514), mRNA | -24.1 | position 1586  target 5' G CUGAAAA C 3'  GCU GUGUGCUGCU  CGG UACACGACGA  miRNA 3' A UUAUAAC U 5' |
|  | NM_001200091.1 | Ictalurus punctatus follicle-stimulating hormone receptor (LOC100304505), mRNA | -27.2 | position 1901  target 5' A G U 3'  UGCCAA GUGCUGCU  ACGGUU CACGACGA  miRNA 3' AUAACUA U 5' |
|  | NM_001200108.1 | Ictalurus punctatus ribosomal protein L8 (LOC100304524), mRNA | -28.8 | position 505  target 5' A U U G 3'  GCUG UGUUGGUG UGUUGCUG  CGGU AUAACUAC ACGACGAU  miRNA 3' A U 5' |
|  | NM_001201279.1 | Ictalurus punctatus small subunit of serine palmitoyltransferase a (sspta), mRNA | -23.3 | position 459  target 5' A C G 3'  GUCAA UGGUGU CUGUUA  CGGUU ACUACA GACGAU  miRNA 3' A AUA C 5' |
|  | NM_001200137.1 | Ictalurus punctatus ribosomal protein SA (rpsa), mRNA | -26.6 | position 850  target 5' C C G C 3'  GCUGG AUUGA GCUGCUA  CGGUU UAACU CGACGAU  miRNA 3' A A ACA 5' |
